# A model of lymphocryptovirus-associated post-transplant lymphoproliferative disorder in immunosuppressed Mauritian cynomolgus macaques

**DOI:** 10.1371/journal.ppat.1012644

**Published:** 2024-11-11

**Authors:** Helen L. Wu, Whitney C. Weber, Courtney M. Waytashek, Carla D. Boyle, Jason S. Reed, Katherine B. Bateman, Hannah K. Fisher, Yan Chen, Kimberly Armantrout, Tonya Swanson, Christine Shriver-Munsch, Mina Northrup, Miranda Fischer, Sreya Biswas, John Templon, Angela Panoskaltsis-Mortari, Benjamin J. Burwitz, Amanda L. Johnson, Lois Colgin, Anne D. Lewis, Jeremy V. Smedley, Michael K. Axthelm, Rebecca Skalsky, Gabrielle Meyers, Richard T. Maziarz, Erik Mittra, Melissa Berg, Jeffrey J. Stanton, Jonah B. Sacha

**Affiliations:** 1 Oregon National Primate Research Center; Oregon Health & Science University; Beaverton, Oregon, United States of America; 2 Division of Blood and Marrow Transplantation, Department of Pediatrics; University of Minnesota; Minneapolis, Minnesota, United States of America; 3 Blood and Marrow Transplant Program, Knight Cancer Institute; Oregon Health & Science University; Portland, Oregon, United States of America; 4 Division of Nuclear Medicine and Molecular Imaging; Oregon Health & Science University; Portland, Oregon, United States of America; National Cancer Institute, UNITED STATES OF AMERICA

## Abstract

Immunocompromised individuals are at risk for developing lymphocryptovirus-associated lymphoproliferative diseases, such as Epstein Barr virus (EBV)-associated B cell lymphomas and post-transplant lymphoproliferative disorder (PTLD). We previously reported development of cynomolgus lymphocryptovirus (CyLCV)-associated PTLD in Mauritian cynomolgus macaques (MCMs) undergoing hematopoietic stem cell transplantation (HSCT), which mirrored EBV-PTLD in transplant patients. Here, we sought to develop a MCM model of lymphocryptovirus-associated lymphoproliferative disease in immunosuppressed MCMs without HSCT. Five simian immunodeficiency virus (SIV)-infected, CD8α+ cell-depleted MCMs received an infusion of autologous B-lymphoblastoid cells transformed with CyLCV, followed by varying degrees of immunosuppression. Four of five infused macaques developed masses coincident with increasing CyLCV plasma viremia, and necropsies confirmed the presence of multicentric lymphomas, which most commonly manifested in lymph nodes, gastrointestinal tract, adrenal glands, and pancreas. Affected tissues harbored neoplastic lymphocytes double-positive for CD20 and CyLCV EBNA2 antigen, large frequencies of proliferating B cells, and high levels of cell-associated CyLCV DNA. In addition, longitudinal 18F-fluorodeoxyglucose positron-emission tomography (18F-FDG PET) of one MCM successfully detected lymphoproliferative disease in the adrenal glands prior to clinical signs of disease. These data demonstrate successful induction of lymphocryptovirus-associated PTLD-like disease in 4 of 5 MCMs, and thus support the use of MCMs as a preclinical NHP model of EBV-associated lymphoproliferative disease that could be employed to test novel diagnostic and therapeutic modalities.

## Author summary

Post-transplant lymphoproliferative disorder (PTLD) is a significant transplant-related complication that typically results from uncontrolled proliferation of lymphocryptovirus-infected B cells in the absence of sufficient immune surveillance. We sought to develop a nonhuman primate model of PTLD-like disease by infusing lymphocryptovirus-infected B cells into immunosuppressed Mauritian cynomolgus macaques. This strategy successfully induced lymphocryptovirus-associated lymphoproliferative disease in 4 of 5 studied macaques, who developed LCV+ B cell lymphomas. While further studies are needed to determine the optimal immunosuppressive regimen to universally trigger disease, our findings lay the foundation for a physiologically relevant macaque model of lymphocryptovirus-associated PTLD that can be employed to test new therapies.

## Introduction

Lymphocryptoviruses are a genus of ubiquitous, persistent gammaherpesviruses with the ability to latently infect and transform host B cells [1]. The human lymphocryptovirus Epstein-Barr virus (EBV) infects over 90% of adults globally, typically resulting in life-long, asymptomatic infections. However, in immunocompromised individuals such as people living with HIV or undergoing transplantation, EBV is associated with numerous B cell malignancies and post-transplant lymphoproliferative disorder (PTLD) [2].

PTLD is a spectrum of disorders ranging from B cell hyperplasia to monoclonal B cell lymphoma, and represents the most common malignancy following transplantation [3]. Over 90% of PTLD cases are caused by uncontrolled proliferation of EBV-infected B cells in the absence of sufficient immune surveillance, particularly T cell immunity [4–6]. Accordingly, risk of EBV-associated PTLD is predominantly related to the degree of T cell depletion or impairment [7–15]. In the clinic, EBV-PTLD can be challenging to diagnose and manage among the milieu of other transplant-related complications. The gold standard for diagnosis of EBV-PTLD is detection of EBV EBNA2 antigen-positive lymphoproliferation in tissue biopsy, which requires knowledge of disease location and can be challenging to obtain. Thus, 18F-fluorodeoxyglucose positron-emission tomography (18F-FDG PET) has recently been adopted as a non-invasive diagnostic imaging tool with high sensitivity and specificity for detecting PTLD in transplant patients, in which elevated uptake of radiolabeled glucose analog FDG denotes glucose hypermetabolism indicative of malignancy [16,17]. Treatment of EBV-PTLD is also challenging as no antiviral has proven effective, and thus existing treatments consist of B cell depletion, reduction of immunosuppression, chemotherapy, and T cell infusions, none of which are universally effective and all of which must be weighed against risk of other complications such as opportunistic infections, graft rejection, and graft-versus-host disease [18–20]. Thus, a physiologically relevant animal model would be invaluable for development and evaluation of new therapeutic strategies for EBV-PTLD.

Simian lymphocryptoviruses are highly prevalent among nonhuman primates (NHPs) and closely mirror EBV in infection, pathogenesis, and immunity [21,22], and thus NHPs represent promising animal models for EBV disease. Cynomolgus lymphocryptovirus (CyLCV) possesses a viral gene repertoire identical to EBV, immortalizes host B cells, and is associated with B cell lymphomas in immunocompromised hosts [23–27]. We, and others, previously reported development of cynomolgus lymphocryptovirus (CyLCV)-associated PTLD in cynomolgus macaques undergoing xenotransplantation, solid organ transplantation, and hematopoietic stem cell transplantation (HSCT) that closely mirrors EBV-PTLD [6,28–32].

Here, we first document the challenges in diagnosis and resolution of CyLCV-PTLD in two HSCT recipient Mauritian cynomolgus macaques (MCMs), which parallel the challenges in management of EBV-PTLD in the clinic. In order to address challenges in diagnosis and treatment of lymphocryptovirus-associated PTLD, we next sought to develop an experimental NHP model of lymphocryptovirus-associated lymphoproliferative disease in MCMs without resource-intensive transplantation. To this end, we used CyLCV harvested from PTLD-experiencing HSCT recipient MCMs to infect and transform healthy MCM B cells *ex vivo*, creating CyLCV-B-lymphoblastoid cell lines (CyLCV-BLCLs). We hypothesized that infusion of autologous CyLCV-BLCLs into simian immunodeficiency virus (SIV)-infected, CD8α+ cell-depleted MCMs would predispose macaques to PTLD-like disease, particularly with additional immunosuppression impacting T cells known to be critical for maintaining EBV control. This strategy successfully induced development of CyLCV+ B cell lymphomas in 4 of 5 MCMs, recapitulating lymphocryptovirus-associated PTLD.

## Results

### Treatment of CyLCV-associated post-transplant lymphoproliferative disease in HSCT recipient MCMs

**Fig 1:**
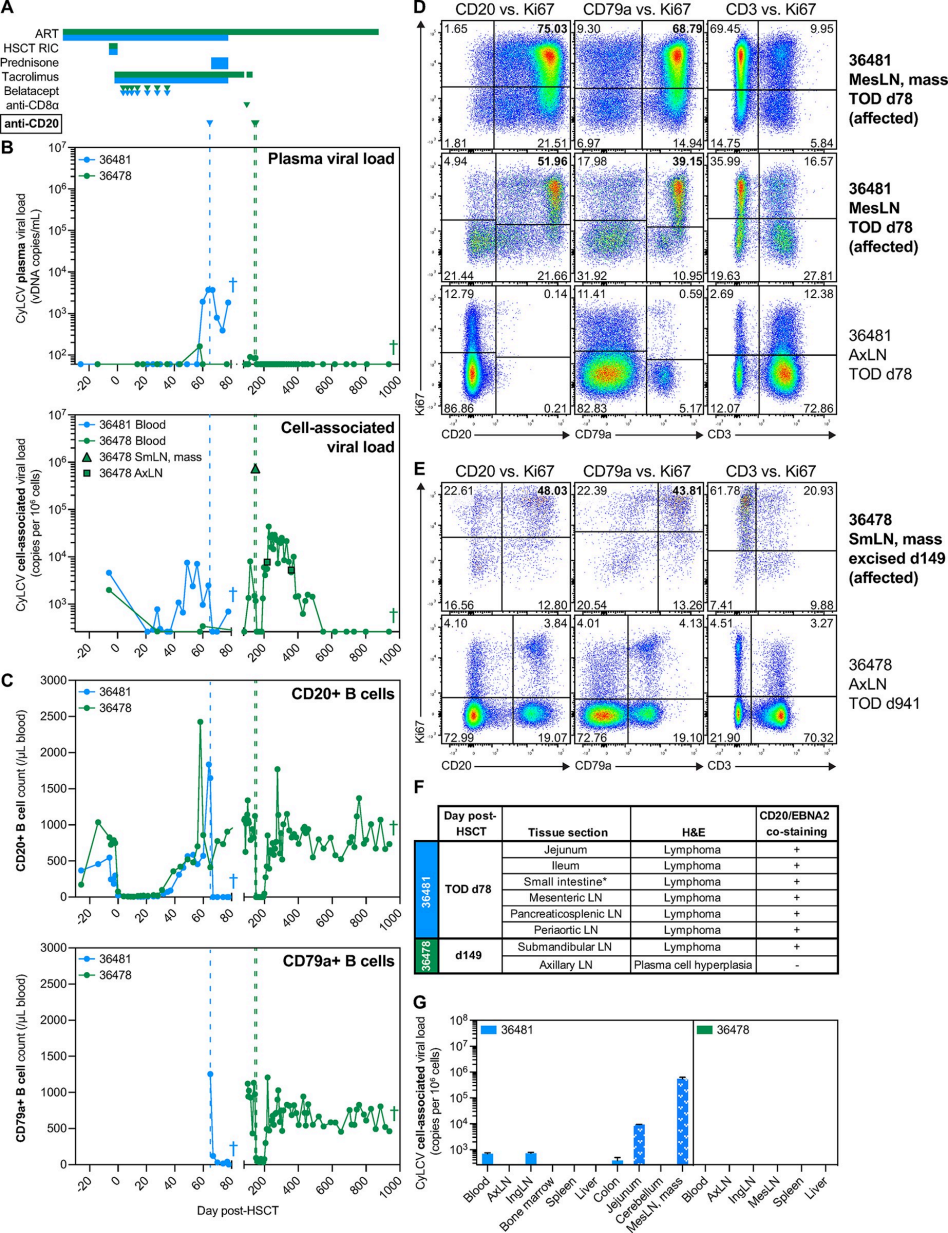
Treatment of CyLCV-associated post-transplant lymphoproliferative disease in HSCT recipient MCMs is challenging. **(A)** Drug regimens with timepoints aligned to graphs in B, C, with colors corresponding to each MCM shown in graphs. **(B)** Longitudinal plasma (top) and cell-associated (bottom) CyLCV DNA viral loads. Limit of quantification (LOQ) = 60 copies/mL for plasma, 260 copies/million cells for cell-associated. Undetectable and below LOQ measurements graphed at the LOQ. Colored vertical dashed lines represent timepoints of anti-CD20 monoclonal antibody administration. Graphs show mean viral loads of two qPCR replicates. **(C)** Longitudinal CD20+ B cell (top) and CD79a+ B cell (bottom) absolute counts in blood. **(D, E)** Representative flow cytometry plots showing intracellular Ki67 staining of B cells and T cells in MCM 36481 (D) and 36478 (E) tissues. Plots are gated on live, CD45+ singlets. Numbers indicate cell frequencies within each quadrant. Bolded tissues represent affected tissues positive for lymphoma. **(F)** Table summarizes tissue sections with signs of lymphoproliferative disease assessed by H&E and presence of cells double-positive for CD20 and EBNA2 assessed by dual immunohistochemistry. *section not specified. **(G)** Cell-associated CyLCV DNA viral loads of necropsy tissues. White patterned bars denote tissues positive for lymphoma. Bars show mean ±SD of two qPCR replicates. Limit of quantification (LOQ) = 260 copies/million cells for cell-associated. Undetectable and below LOQ measurements graphed at the LOQ. Colored crosses next to datapoints indicate time of euthanasia/death for each MCM. ART = antiretroviral therapy, RIC = reduced intensity conditioning, SmLN = submandibular lymph node, AxLN = axillary lymph node, IngLN = inguinal lymph node, MesLN = mesenteric lymph node, TOD = time of death.

We previously reported development of CyLCV-associated PTLD in MCMs undergoing reduced-intensity allogeneic HSCT (alloHSCT) [30]. In two such alloHSCT recipient MCMs, we attempted to resolve LCV disease with therapeutic intervention (Fig 1). Both HSCT recipient MCMs underwent reduced intensity conditioning with T cell depletion (CD3-immunotoxin), single dose chemotherapy busulfan, and total body irradiation for transplantation of fully MHC-matched alloHSCT donor grafts, and received immunosuppression of daily tacrolimus, single dose chemotherapy cyclophosphamide, and seven doses of costimulation blockade belatacept, a high affinity variant of CTLA4-Ig that blocks CD28 costimulatory signaling [30,33,34]. Both HSCT recipient MCMs were SIV-infected, but SIV plasma viremia was fully suppressed by antiretroviral therapy (ART) at the time of HSCT and PTLD manifestation [33].

In the case of HSCT recipient MCM 36481, quantitative PCR detected CyLCV plasma viremia at day 60 post-HSCT, a time when the macaque remained on daily tacrolimus for GVHD prophylaxis (Fig 1A and 1B). CyLCV viremia was accompanied by a large, concomitant increase in blood CD20+ B cell count (Fig 1C), anorexia, and weight loss (S1A Fig), raising the possibility of LCV-PTLD. In an attempt to deplete B cells and prevent further disease development, anti-CD20 depleting antibody was administered the following day (day 65 post-HSCT). However, MCM 36481 concurrently developed a progressive macular rash consistent with cutaneous graft-versus-host disease, and thus prednisone treatment was also initiated at day 65 post-HSCT. Despite initial decreases in plasma and whole blood cell-associated CyLCV viral loads and depletion of blood B cells after anti-CD20 intervention, ultrasound at day 74 post-HSCT revealed an abdominal mass, weight loss continued, and CyLCV viral loads rebounded (Figs 1B, 1C and S1A). Of note, B cell counts were also measured by staining for intracellular B cell marker CD79a as the presence of anti-CD20 depleting antibody can block anti-CD20 clone 2H7 staining. Necropsy at day 78 post-HSCT revealed a large mesenteric lymph node mass and confirmed lymphoma in periaortic, sacral, pancreaticosplenic, and mesenteric lymph nodes and gut-associated lymphoid tissue of the jejunum and ileum, including multifocal invasion of the adipose and connective tissues surrounding lymph nodes. Flow cytometric staining of cell suspensions from necropsy tissues demonstrated that despite effective anti-CD20-mediated depletion of blood B cells in MCM 36481 (Fig 1C), large frequencies of live, proliferating Ki67+ CD20+ and CD79a+ B cells persisted in the mesenteric lymph node mass and other grossly normal mesenteric lymph nodes and tissues (Figs 1D and S1B). In addition, while T cell frequencies within the mesenteric lymph node mass and other mesenteric lymph nodes were dwarfed by B cell frequencies, a large proportion of T cells were Ki67+ (Figs 1D and S1B), suggesting the presence of a T cell response attempting to control disease.

In the second case, HSCT recipient MCM 36478 exhibited a transient low-level blip of CyLCV plasma viremia accompanied by a spike in blood CD20+ B cells at day 58 post-HSCT (Fig 1B and 1C). However, both spontaneously resolved within days, and blood cell-associated CyLCV remained at or below pre-transplant levels through day 100 post-HSCT (Fig 1B). At day 99 post-HSCT, graft rejection was suspected due to rapidly decreasing T cell donor chimerism [33] and thus anti-CD8α depleting antibody was administered, additional donor lymphocytes were infused, and daily tacrolimus was temporarily re-initiated. Approximately 40 days later, after tacrolimus had been discontinued again, MCM 36478 presented with a submandibular mass. A fine needle aspirate revealed the mass was composed of 99% lymphocytes, and mass detection was accompanied by recurrence of low-level CyLCV plasma viremia as well as an increase in blood cell-associated CyLCV (Fig 1B). Due to suspicion of LCV-PTLD, anti-CD20 depleting monoclonal antibody was administered the same day (day 144 post-HSCT) and the mass was surgically excised five days later at day 149 post-HSCT, revealing two distinct but adhered submandibular lymph nodes with lymphoma and extensive necrosis. While the anti-CD20 dose successfully depleted peripheral blood B cells (Fig 1C), low level CyLCV plasma viremia persisted and the excised mass not only harbored large quantities of cell-associated CyLCV DNA but also a substantial frequency of live, proliferating Ki67+ CD20+ B cells (Figs 1B, 1E and S1C). Thus, an additional dose of anti-CD20 was administered at day 154 post-HSCT in an attempt to ablate surviving CyLCV+ B cells. After intervention, plasma CyLCV viremia fell below detectable levels and remained undetectable (Fig 1B). Blood cell-associated CyLCV viral loads transiently decreased, then rebounded as blood B cell counts rebounded and remained above pre-transplant levels for a period of approximately 200 days (Fig 1B and 1C). However, copy numbers of blood cell-associated CyLCV were similar to those measured in unremarkable axillary lymph nodes biopsied at day 219 and 361 post-HSCT, eventually waned to pre-transplant levels, and then fell below detectable levels (Fig 1B). MCM 36478 maintained and eventually gained weight (S1A Fig), and no further signs of LCV disease were observed in the additional ~700 days of monitoring before euthanasia, including approximately 200 days off-ART with active SIV plasma viremia [33]. Planned necropsy of MCM 36478 at day 941 post-HSCT did not reveal any findings suggestive of ongoing or previous lymphoproliferative disease, including unremarkable frequencies of B cells and proliferating Ki67+ B and T cells in necropsy tissues compared to the previously excised submandibular lymph node mass (Figs 1E and S1C).

To further confirm the cellular origin and viral etiology of suspected LCV-PTLD in HSCT recipient MCMs 36481 and 36478, we performed multiplex immunohistochemistry staining for B cell marker CD20 and lymphocryptovirus Epstein-Barr nuclear antigen-2 (EBNA-2) on sections of a variety of diseased necropsy tissues from MCM 36481 and the excised submandibular mass from MCM 36478 (Figs 1F and S1D), using an CyLCV-crossreactive EBV EBNA-2 antibody. The majority of cells within neoplasms were positive for both membrane CD20 and nuclear EBNA-2, demonstrating LCV-associated B cell-lineage lymphoproliferative disease. Finally, we measured cell-associated CyLCV DNA in a variety of necropsy tissues from both MCMs (Fig 1G). The mesenteric lymph node mass from MCM 36481 was highly positive for CyLCV DNA, at a level comparable to that measured in MCM 36478 excised submandibular lymph node mass (Fig 1B and 1G). In contrast, B cell-depleted necropsy tissues from MCM 36481 and all tested necropsy tissues from MCM 36478 contained little to no CyLCV DNA. Together, these findings are consistent with development of CyLCV-PTLD in HSCT recipient MCMs 36481 and 36478, and demonstrate that the diagnostic and treatment challenges for CyLCV-PTLD mirror those reported for EBV-PTLD.

### PTLD-derived CyLCV can be used to generate B-lymphoblastoid cell lines

**Fig 2:**
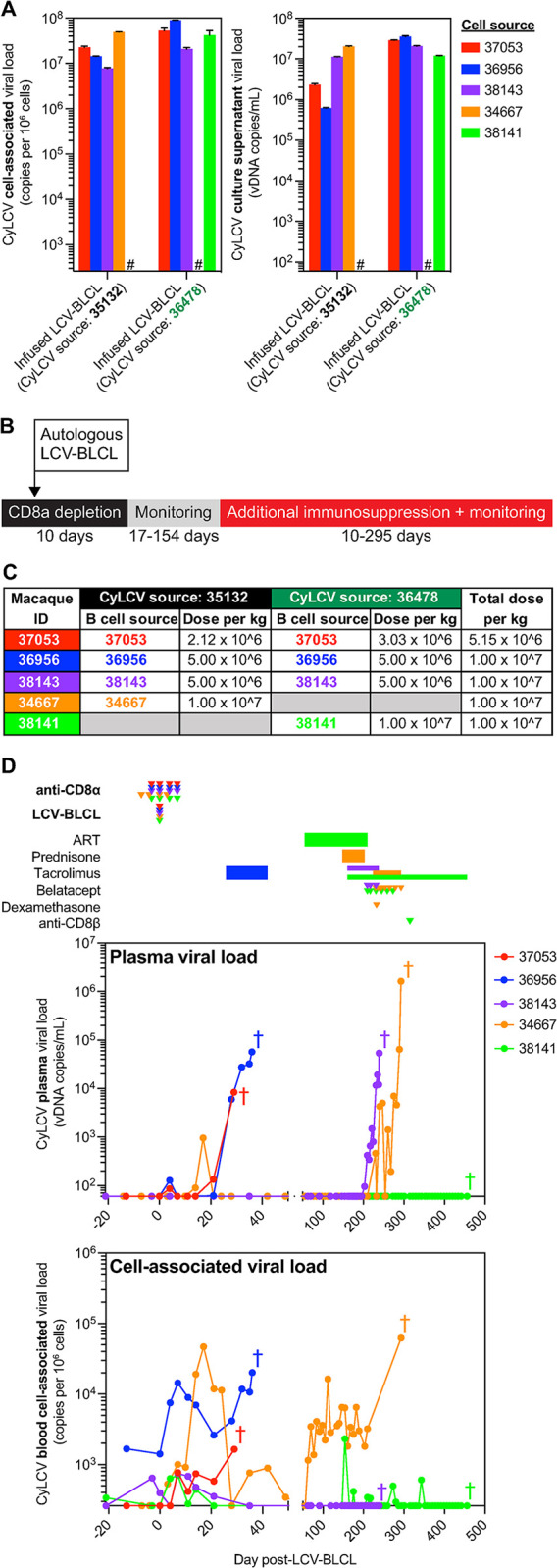
Generation and infusion of CyLCV-BLCLs into immunosuppressed LCV model MCMs. **(A)** Cell-associated (left) and supernatant (right) CyLCV DNA viral loads in *ex vivo*-generated CyLCV-BLCL cultures using CyLCV from PTLD-experiencing HSCT recipient MCM. Bars show mean ±SD of two qPCR replicates. Hashtags (#) indicate CyLCV-BLCL lines not generated. **(B)** Study outline for LCV model MCMs. **(C)** Table summarizing dosing of autologous CyLCV-BLCL infusions into LCV model MCMs. **(D)** Longitudinal plasma (top) and blood cell-associated (bottom) CyLCV DNA viral loads. LOQ = 60 copies/mL for plasma, 260 copies/million cells for blood cells. Graphs show mean viral loads of two qPCR replicates. Drug regimens and CyLCV-BLCL infusion timepoints indicated above graphs with colors corresponding to each MCM shown in graphs. Colored crosses next to datapoints indicate time of euthanasia/death for each MCM.

EBV was first discovered and isolated from a Burkitt’s lymphoma culture [35], and has historically been utilized to transform and immortalize human B cells *ex vivo*, generating B-lymphoblastoid cell lines (BLCLs) [36]. Similarly, simian LCV-transformed B cells can be cultured from NHP tissues, and simian LCVs such as baboon LCV are commonly used to create NHP BLCLs *ex vivo* [23,37,38]. We previously reported that cultures of single cell suspensions harvested from a CyLCV-PTLD-experiencing HSCT recipient MCM in simple R10 medium without any exogenous cytokines or growth factors resulted in CyLCV+ BLCLs that could be maintained indefinitely [30]. Thus, we cultured single cell suspensions harvested from diseased tissues of HSCT recipient MCMs 36481 and 36478 in R10, which resulted in expansion of BLCLs. In all CyLCV-PTLD tissue-derived BLCL cultures, both cells and clarified culture supernatants tested positive for CyLCV DNA (S1E Fig), suggesting CyLCV-PTLD tissue-derived BLCLs were actively producing CyLCV. Indeed, *ex vivo* addition of clarified, filtered culture supernatant from CyLCV-PTLD tissue-derived BLCL to primary peripheral blood CD20+ B cells from other healthy MCM created new CyLCV+ BLCL lines, termed “CyLCV-BLCL” (Fig 2A). We created eight new CyLCV-BLCL lines using primary peripheral blood B cells from five MCMs and clarified, filtered CyLCV-containing supernatants from two primary CyLCV-PTLD tissue-derived BLCL cultures: inguinal lymph node cells from MCM 35132 [30] and excised submandibular lymph node mass from MCM 36478 (Fig 2A). MCM 35132 underwent a very similar allogeneic HSCT regimen to MCM 36478 and developed CD20+EBNA2+ lymphomas in lymph nodes, spleen, liver, kidney, and adrenal glands [30], and cultured inguinal lymph node cells from necropsy harbored a similar level of cell-associated CyLCV as the cultured excised submandibular lymph node mass cells from MCM 36478 (S1E Fig). Whole genome sequencing of supernatant virion DNA from both the 35132 inguinal lymph node culture and 36478 submandibular lymph node culture confirmed intact, full-length CyLCV genomes with no major insertions or deletions, and aligned to previously published CyLCV (NC_055142) with 97.9% and 98.1% pair-wise identify, respectively (Genbank accession numbers PQ385619, PQ385620). Similar to primary CyLCV-PTLD tissue-derived BLCL, *ex vivo*-generated CyLCV-BLCL contained cell-associated CyLCV DNA, secreted CyLCV DNA into culture supernatant, and could be cultured indefinitely in simple medium (Fig 2A). Thus, CyLCV from PTLD-experiencing HSCT recipient MCMs could be utilized to generate CyLCV-BLCLs from healthy MCM.

### Mauritian cynomolgus macaques infused with autologous CyLCV-BLCLs develop LCV-PTLD-like disease

Due to the challenges in treating EBV-PTLD in humans and CyLCV-PTLD in MCMs, we sought to develop a streamlined model of LCV-associated lymphoproliferative disease in MCMs that did not require transplantation but could be utilized to test new treatments. We hypothesized that infusing autologous CyLCV-BLCLs into SIV-infected, CD8α+ cell-depleted MCMs would predispose macaques to PTLD-like disease, particularly with addition of T cell-targeting immunosuppression commonly employed in transplantation. Thus, we performed a study with five SIV-infected, CyLCV-seropositive MCMs from which we had generated CyLCV-BLCL *ex vivo* using CyLCV-containing supernatants from primary 35132 and 36478 CyLCV-BLCL cultures (Fig 2A). The five study MCMs received four doses of anti-CD8α depleting monoclonal antibody in an attempt to prevent rapid clearance of infused autologous CyLCV-BLCL by antiviral CD8+ T cells and NK cells (Figs 2B and S2). During the period of CD8α+ cell depletion, we infused autologous CyLCV-BLCL intravenously at 0.5-1x10^7^ cells/kg (Fig 2C). Two macaques were infused with a single autologous CyLCV-BLCL line transformed with CyLCV-containing supernatant from either the 35132 inguinal lymph node culture (MCM 34667) or the 36478 submandibular lymph node culture (MCM 38141). In contrast, three macaques (MCMs 37053, 36956, 38143) were infused with approximately equal ratios of two autologous CyLCV-BLCL lines, one line transformed with CyLCV-containing supernatant from the 35132 inguinal lymph node culture and one line transformed with CyLCV-containing supernatant from the 36478 submandibular lymph node mass culture. Infused CyLCV-BLCL lines expressed surface CD20 at variable levels (S3A Fig) and proliferated at similar rates *in vitro* (S3B Fig). We monitored for development of LCV lymphoproliferative disease by plasma and blood cell-associated CyLCV viral loads, physical examinations and ultrasound, and in one case 18F-FDG PET.

All MCMs were SIVmac239-viremic and CyLCV-seropositive, but did not have active CyLCV plasma viremia at study day 0 (Figs 2D and S4A). In all macaques, anti-CD8α administration successfully depleted blood CD8+ T cells and NK cells prior to CyLCV-BLCL infusion, resulting in increased SIV plasma viremia (S4A and S4B Fig). Shortly after LCV-BLCL infusion, blood cell-associated CyLCV viral loads increased in four of the five MCMs (37053, 36956, 34667, 38141) and transient blips of CyLCV plasma viremia occurred in three of the five MCMs (37053, 36956, and 34667) (Fig 2D). CyLCV plasma viremia returned in MCM 37053 within 3 weeks of CyLCV-BLCL infusion. Increasing CyLCV viremia in MCM 37053 was accompanied by development of an abdominal mass, detected by ultrasound at day 25 post-CyLCV-BLCL infusion, prompting euthanasia at day 29.

The remaining four MCM did not show any sign of disease progression by three weeks post-infusion and thus received varying additional immunosuppression, including calcineurin inhibitor tacrolimus, corticosteroids prednisone and dexamethasone, T cell costimulation blockade monoclonal antibody belatacept, and/or anti-CD8β depleting monoclonal antibody (Figs 2D and S2). MCM 36956 received daily tacrolimus beginning at day 26 post-CyLCV-BLCL infusion, which was followed by a rapid and continual increase in CyLCV plasma viremia and ultrasound detection of round, variable-sized, hypoechoic lesions throughout the liver on day 35, leading to euthanasia the following day at day 36. MCM 38143 remained mostly aviremic on tacrolimus monotherapy (day 161 to 210), but CyLCV plasma viremia rapidly increased during belatacept dosing between days 210 and 231, and an abdominal mass was detected at day 235, leading to euthanasia at day 239. MCM 34667 remained aviremic despite 55 days of prednisone treatment and taper, but rapidly developed CyLCV plasma viremia upon initiation of daily tacrolimus at day 224, which continued to trend upward after immunosuppression intensification with dexamethasone and belatacept between days 230 and 293. This was accompanied by anorexia and weight loss (S4C Fig). At day 293, MCM 34667 presented with a firm immobile mass in the right upper abdomen and gas distention in the left upper quadrant, and ultrasound confirmed several masses near the right kidney with a large amount of free fluid in the abdomen, prompting euthanasia later the same day.

In contrast to the other four study macaques, MCM 38141 remained undetectable for CyLCV plasma viremia throughout the study (Fig 2D). Of note, MCM 38141 was the only animal temporarily treated with daily combination anti-retroviral therapy (ART) due to severe blood CD4+ T cell depletion and concern that an SIV/AIDS-associated complication unrelated to CyLCV would develop and lead to premature euthanasia (S4A Fig). Daily ART successfully suppressed SIV viremia and restored CD4+ T cell counts (S4A Fig). However, daily tacrolimus for almost 50 days was insufficient to trigger CyLCV-associated lymphoproliferative disease in MCM 38141 while SIV viremia was suppressed. Thus, we decided to discontinue ART prior to the first belatacept dose, while continuing daily tacrolimus. However, SIV plasma viremia did not return for about 13 weeks after ART discontinuation and six doses of belatacept failed to induce any signs of disease. Shortly after rebound of SIV plasma viremia at day 301, we made a final attempt to trigger CyLCV-associated disease with CD8β+ cell depletion. Administration of anti-CD8β depleting monoclonal antibody at day 315 effectively depleted CD8β+ T cells and was followed by a large increase in SIV plasma viremia and CD4+ T cell depletion (S4A and S4B Fig), but MCM 38141 continued to control CyLCV (Fig 2D). Thus, despite a total of nearly 300 days of daily tacrolimus treatment, ART discontinuation, SIV rebound and associated CD4+ T cell depletion, belatacept, and CD8β+ cell depletion, MCM 38141 exhibited only transient, low-level blood cell-associated CyLCV and no clinical signs of LCV disease throughout the study until planned euthanasia at day 456.

**Fig 3:**
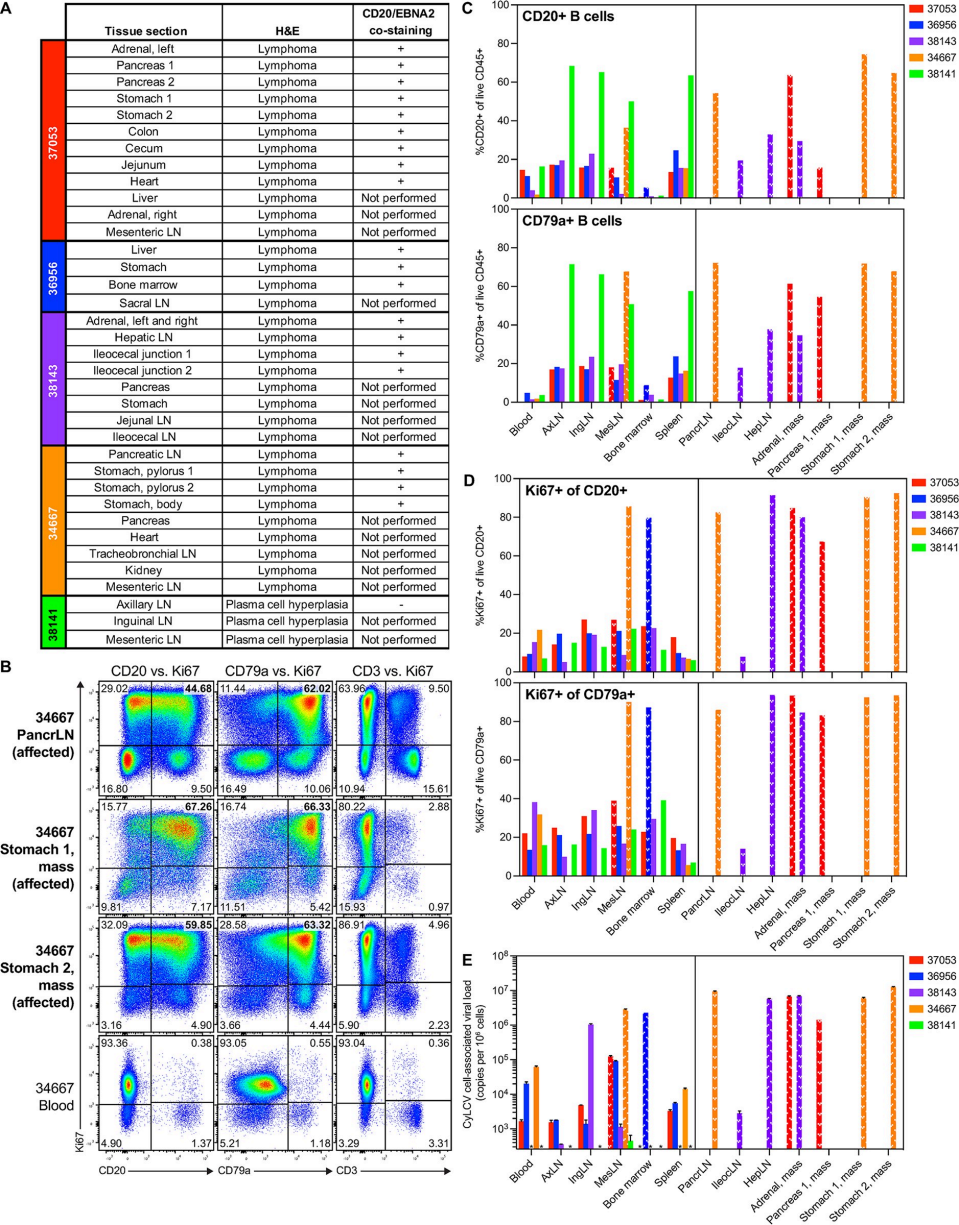
Majority of CyLCV-BLCL-infused, immunosuppressed LCV model MCMs develop CyLCV-associated lymphoproliferative disease. **(A)** Table summarizes tissue sections with signs of lymphoproliferative disease assessed by H&E staining and presence of cells double-positive for CD20 and EBNA2 assessed by dual immunohistochemistry. **(B)** Representative flow cytometry plots showing intracellular Ki67 staining of B cells and T cells in MCM 34667 necropsy tissues. Plots are gated on live, CD45+ singlets. Numbers indicate cell frequencies within each quadrant. Bolded tissues represent affected tissues positive for lymphoma. **(C, D)** Summary graphs of flow cytometry staining of LCV model MCM necropsy tissues. Frequencies of CD20+ B cells and CD79a+ B cells among live CD45+ cells (C). Frequencies of Ki67+ cells among live CD20+ B cells and CD79a+ B cells (D). White patterned bars denote affected tissues positive for lymphoma. Bars not shown indicate samples not assayed. **(E)** Cell-associated CyLCV DNA viral loads of necropsy tissues. White patterned bars denote affected tissues positive for lymphoma. Bars show mean ±SD of two qPCR replicates. LOQ = 260 copies/million cells. Asterisks (*) indicate samples that measured below LOQ. Other bars not shown indicate samples not assayed. PancrLN = pancreatic lymph node, AxLN = axillary lymph node, IngLN = inguinal lymph node, MesLN = mesenteric lymph node, IleocLN = ileocecal lymph node, HepLN = hepatic LN.

Gross and microscopic (hematoxylin and eosin, H&E) examination of necropsy tissues revealed multicentric lymphomas in the four MCMs that developed masses (37053, 36956, 38143, 34667) (Fig 3A). Lymphomas developed in diverse nodal and extranodal sites, including the gastrointestinal tract, liver, kidney, adrenal glands, pancreas, and heart. Tumors in affected MCMs were notable for the frequent presence and degree of central necrosis and hemorrhage and the presence of intravascular rafts of neoplastic lymphocytes. To confirm suspected LCV lymphoproliferative disease, we performed multiplex CD20/EBNA-2 immunohistochemistry staining on sections of necropsy tissues with signs of lymphoproliferative disease. Similar to LCV-PTLD-experiencing HSCT recipient MCMs, all tested lymphomas from LCV model study macaques contained large frequencies of cells positive for both CD20 and EBNA-2 (Figs 3A and S5). Flow cytometric staining of cell suspensions harvested from diseased tissues showed large frequencies of CD20+ and CD79a+ B cells, which were highly positive for proliferation marker Ki67 (up to 94% Ki67+), relative to blood and unremarkable tissues from the same MCM (Fig 3B–3D). In the vast majority of diseased tissues, B cell frequencies overwhelmed T cell frequencies, but existing T cells were also highly positive for Ki67 (S6A Fig), similar to CyLCV-PTLD tissues from HSCT recipient MCMs 36481 and 36478. Also consistent with CyLCV-associated disease, substantial amounts of cell-associated CyLCV DNA were detected in affected necropsy tissues, while unaffected tissues had little to no detectable CyLCV DNA (Fig 3E). Finally, culture of isolated CD20+ cells from 37053, 36956, 38143, and 34667 affected necropsy tissues in simple R10 medium led to outgrowth of primary BLCL cultures positive for CyLCV DNA in both cells and supernatant (S6B Fig). In contrast, isolated CD20+ cells from unaffected axillary lymph nodes of 37053 harbored cell-associated CyLCV DNA, but did not secrete detectable levels of CyLCV DNA into culture supernatant. Whole genome sequencing of supernatant virion DNA from 37053 adrenal gland, 36956 bone marrow, and 38143 adrenal gland BLCL cultures confirmed intact, full-length CyLCV genomes with no major insertions or deletions (Genbank accession numbers PQ385621, PQ385622, PQ385623). Together, these findings are consistent with development of B cell-lineage LCV-lymphoproliferative disease resembling EBV/LCV-PTLD in four of five LCV model macaques.

In contrast to the other four MCMs and consistent with the longitudinal data, CyLCV-associated lymphoproliferative disease could not be confirmed in MCM 38141 after examination of necropsy tissues. While multiple lymph nodes exhibited plasma cell hyperplasia, a tested axillary lymph node section was negative for EBNA-2 (Fig 3A). Flow cytometry staining showed elevated B cell frequencies in axillary, inguinal, and mesenteric lymph nodes and spleen of MCM 38141, similar to frequencies observed in masses from the other four diseased MCMs (Fig 3C). However, B and T cell Ki67 expression in these tissues was unremarkable compared to that observed in diseased MCM tissues (Figs 3D and S6A). Further, CyLCV DNA was undetectable in MCM 38141 necropsy tissues with the exception of mesenteric lymph nodes, which harbored a low-level of CyLCV DNA consistent with a healthy CyLCV-seropositive MCM (Fig 3E). Finally, isolated CD20+ cells from 38141 axillary and inguinal lymph nodes collected at necropsy did not expand when cultured in simple R10 medium. While cell-associated CyLCV was detectable in the inguinal lymph node culture, it was not detectable in the axillary lymph node culture (S6B Fig). Further, neither axillary and inguinal lymph node cultures secreted detectable levels of CyLCV DNA into culture supernatants. Together, these results suggest failure to induce bona fide CyLCV-associated lymphoproliferative disease in one of five LCV model macaques.

### 18F-fluorodeoxyglucose (FDG) PET imaging can be used to detect and monitor LCV-associated lymphoproliferative disease in macaques

**Fig 4:**
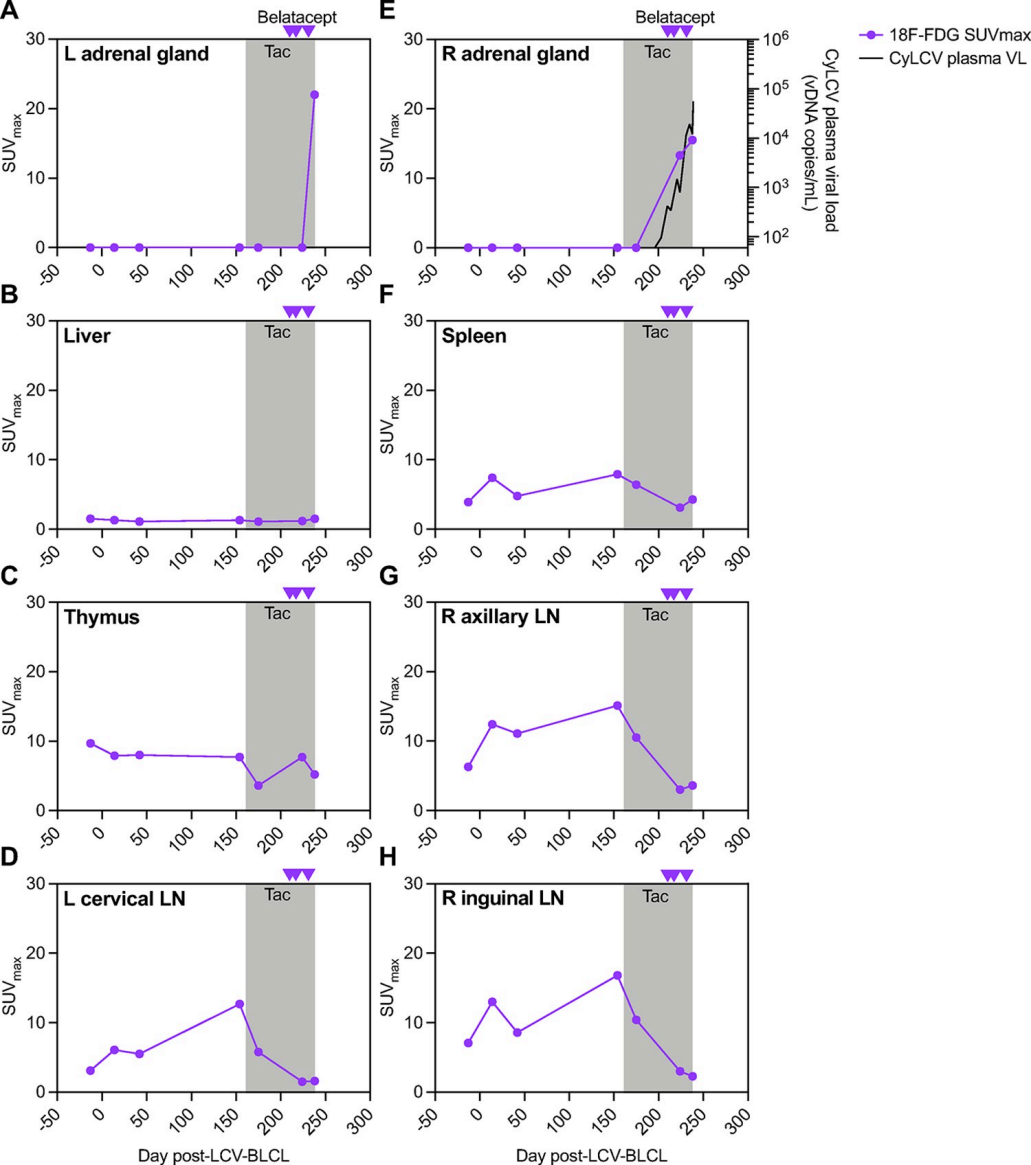
18F-FDG PET of model MCM 38143. **(A-H)** Longitudinal maximum standardized uptake values (SUVmax) for 18F-FDG in various tissues from scans of model MCM 38143. Gray boxes denote the period of daily tacrolimus (tac) treatment. Purple arrowheads denote timepoints of belatacept administration. Longitudinal 38143 plasma CyLCV DNA viral loads are overlayed on right adrenal gland graph (E, right axis), line shows mean viral load of two qPCR replicates. LOQ = 60 copies/mL plasma. Undetectable and below LOQ measurements graphed at the LOQ. L = left, R = right, LN = lymph node.

We also sought to determine if 18F-FDG PET could be used to detect and monitor LCV-associated lymphoproliferative disease in macaques. To this end, LCV model study MCM 38143 underwent computed tomography (CT) coupled with 18F-FDG PET at baseline and various timepoints after CyLCV-BLCL infusion and immunosuppression. In order to assess changes in FDG uptake over the course of the study, we monitored maximum standardized uptake values (SUVmax) for 18F-FDG in various tissues from each scan (Fig 4). No substantial changes in 18F-FDG uptake were observed through day 42 post-CyLCV-BLCL infusion, consistent with lack of CyLCV plasma viremia nor overt disease during this time period (Figs 2D and 4A-4H). A scan at day 154, one week prior to tacrolimus initiation, showed mild elevations in 18F-FDG uptake in lymph nodes, but SUVmax values returned to baseline after the first 2 weeks of daily tacrolimus treatment at day 175. Belatacept administration at days 210 and 217 triggered increasing CyLCV plasma viremia (Figs 2D and 4E), but no clinical signs of disease were observed by physical examination or abdominal ultrasound through day 221. In contrast, 18F-FDG PET at day 224 showed increasing 18F-FDG uptake (SUVmax 13.3) in the right adrenal gland consistent with a mass (Fig 4E). After a third belatacept dose at day 231, abdominal ultrasound on day 235 detected an abdominal mass, and 18F-FDG PET scan on day 238 showed an increase in the size and FDG uptake of the right adrenal gland lesion (SUVmax 15.5), as well as a lesion with increased FDG uptake in the left adrenal gland (SUVmax 22) (Fig 4A and 4E). As described above, necropsy at day 239 confirmed bilateral CyLCV-associated lymphoproliferative disease in the adrenal glands of MCM 38143 (Fig 3A), demonstrating that increased FDG uptake observed via 18F-FDG PET represented manifestation and progression of LCV-associated lymphoproliferative disease.

## Discussion

Together, the data reported here support the use of CyLCV-infected cynomolgus macaques as a promising, physiologically relevant NHP model of EBV-PTLD. To begin, we demonstrated that the challenges in diagnosis and treatment of CyLCV-PTLD in HSCT recipient MCMs mirror those for EBV-PTLD. First, there is a lack of non-invasive biomarker assays to reliably predict EBV-PTLD [19]. While detection of EBV plasma viral DNA is correlated with risk of lymphoproliferative disease, it can be present in healthy individuals, absent in affected individuals, and is not always specific for disease onset [10,39–41]. We observed similar variability in CyLCV-PTLD-experiencing HSCT recipient MCMs; MCM 36481 had persistent, high CyLCV plasma viral loads (>3,000 copies/mL), while MCM 36478 had transient, barely detectable CyLCV plasma viremia (<200 copies/mL). Second, management of EBV-PTLD is challenging in the context of other transplant-related complications such as GVHD, for which treatment often requires intensified immunosuppression that may exacerbate infectious disease complications. Indeed, HSCT recipient MCM 36481 developed cutaneous GVHD at the same time as suspected CyLCV-PTLD, and thus progression of CyLCV-PTLD was risked in favor of GVHD treatment with immunosuppressant prednisone. Third, while adoption of anti-CD20 depleting monoclonal antibody rituximab has improved prognosis for EBV-PTLD, rituximab is not universally effective, and timing of treatment remains a matter of debate [18,20,42–46]. In HSCT recipient MCMs, we preemptively treated with a macaque-ized version of rituximab, prior to confirmed diagnosis of CyLCV+ lymphoma, and observed varied success. Thus, overall, management of CyLCV-PTLD in MCMs recapitulates key challenges of managing EBV-PTLD in the clinic.

While we previously reported substantial incidence (~30%) of CyLCV-PTLD in MCMs after allogeneic HSCT, performing allogeneic HSCT is expensive and intensive, including total body irradiation, chemotherapy, and collection and infusion of MHC-matched HSC-containing grafts in addition to post-transplant immunosuppression. Thus, here we developed a substantially less intensive, streamlined method to induce PTLD-like disease in SIV+ MCMs using a combination of antibody-mediated CD8α+ cell depletion and infusion of autologous CyLCV-BLCL, paired with up to four additional immunosuppressants commonly employed in transplantation. This strategy successfully induced CyLCV-associated lymphoproliferative disease that closely resembled EBV/CyLCV-PTLD in 4 of 5 macaques. Disease was characterized by CyLCV plasma viremia and development of multifocal CyLCV DNA+ and antigen+ B cell-lineage lymphomas at nodal and extranodal tissue sites, most commonly in lymph nodes (4/4), stomach (4/4), adrenal glands (3/4), and pancreas (3/4). B cells within diseased tissues were highly positive for proliferation marker Ki67 (up to 94% Ki67+), and *ex vivo* culture of diseased tissue CD20+ B cells led to outgrowth of primary CyLCV-BLCLs that harbored CyLCV DNA within cells and in culture supernatant.

Despite successful induction of LCV disease in four MCMs, one MCM (38141) failed to develop severe LCV disease even after prolonged immunosuppression. This could be due to a number of factors, but there are two notable differences between MCM 38141 and the other four model macaques. First, MCM 38141 received daily antiretroviral therapy for a portion of the study, which suppressed SIV viremia and restored CD4+ T cell count, and thus perhaps this macaque did not achieve the necessary degree of immunosuppression to trigger uncontrolled LCV+ B cell proliferation and lymphomagenesis. Indeed, CD4+ T cells can inhibit EBV-induced B cell proliferation [47], and cytotoxic EBV-specific CD4+ T cells have been reported [48]. Second, the source of the CyLCV used to immortalize the infused CyLCV-BLCLs differed between MCM 38141 and the other four LCV model MCMs (Fig 2C). The infusion of 38141 CyLCV-BLCL contained a single CyLCV-BLCL line immortalized with CyLCV sourced from HSCT recipient MCM 36478, whereas the CyLCV-BLCL infused into the other 4 MCMs contained some CyLCV-BLCL immortalized with CyLCV sourced from HSCT recipient MCM 35132. Thus, perhaps CyLCV strain-specific differences translated to differences in pathogenicity *in vivo*. In-depth sequencing analysis of CyLCV strains from a large number of lymphomas from HSCT recipient and LCV model MCMs may identify CyLCV polymorphisms linked to increased pathogenicity. Indeed, substantial amino acid divergence has been reported for viral proteins among EBV and rhesus lymphocryptovirus (RhLCV) strains, and one EBV EBNA-2 sequence is more efficient at immortalizing B cells [49,50].

Overall, the data reported here suggest that the degree of immunosuppression necessary to trigger lymphocryptovirus-associated lymphoproliferative disease may differ among individuals, and that likely multiple combinations of T cell immunosuppression, whether virus- or drug-mediated, are able to achieve the required threshold of immunosuppression for disease. In the small group of animals studied here, lymphocryptovirus-PTLD-like disease was triggered without any additional immunosuppression (n = 1, MCM 37053), after tacrolimus treatment alone (n = 1, MCM 36956), after combination tacrolimus and belatacept (n = 1, MCM 38143), and after combination tacrolimus, belatacept, and dexamethasone (n = 1, MCM 34667). In the two cases with most rapid disease onset with no or minimal additional immunosuppression (MCMs 37053 and 36956), macaques exhibited >3 log higher SIV plasma viral loads as well as lower blood CD4+ T cell counts prior to study initiation, suggesting a higher level of SIV-induced immunosuppression reduced the need for additional drug-mediated immunosuppression to trigger lymphomagenesis. In the latter case of MCM 34667, belatacept and dexamethasone were delivered during the same time period, and thus we cannot determine if one or both were responsible for lymphomagenesis. However tacrolimus, either alone or in combination with belatacept, led to CyLCV plasma viremia in 3 of 5 MCMs, including MCM 34667 before dexamethasone treatment. Indeed, HSCT recipient MCMs that developed CyLCV-PTLD received a combination of tacrolimus and either belatacept or closely related abatacept [30], and tacrolimus monotherapy was associated with development of PTLD in cynomolgus macaques undergoing facial allograft transplantation [31]. Both tacrolimus and belatacept inhibit T cell activation, through calcineurin inhibition or co-stimulation blockade respectively [34,51], and thus likely weaken T cell-mediated control of LCV-infected B cell proliferation. Calcineurin inhibitor cyclosporine is also associated with PTLD in cynomolgus macaques undergoing renal transplantation and xenotransplantation [28,32]. Further studies with additional macaques with consistent pre-existing SIV viremia and defined timing of step-wise drug-mediated immunosuppression, paired with additional readouts of antiviral T cell functionality and monitoring by 18F-FDG imaging, would help define the threshold of immunosuppression necessary to trigger lymphocryptovirus-associated lymphoproliferative disease and ascertain the optimal immunosuppressive regimen for consistent lymphomagenesis.

To our knowledge, this is the first report describing the use of 18F-FDG imaging to identify and monitor lymphocryptovirus-associated lymphoproliferative disease in NHPs. Here, 18F-FDG SUVmax values in diseased adrenal glands of MCM 38143 were similar to those observed in EBV-PTLD patients [16] and reliably identified sites of CyLCV lymphoproliferative disease prior to clinical disease manifestation. Thus, while additional macaque studies are needed to confirm these findings, 18F-FDG imaging represents a promising tool for rapid diagnosis of lymphoproliferative disease and may also be useful for grading disease severity, monitoring progression of disease, and assessment of therapeutic efficacy. Indeed, non-invasive monitoring with 18F-FDG PET would be hugely advantageous for NHP models that aim to evaluate therapeutic interventions but exhibit some heterogeneity in disease timing and outcome, like the MCM model reported here. For example, 18F-FDG PET monitoring could be used on an individual basis to detect and measure LCV lymphoproliferative disease, prompt therapeutic intervention once a pre-determined 18F-FDG SUVmax is exceeded, and then measure therapeutic response longitudinally after treatment.

Rhesus macaques represent an alternative NHP model for EBV-associated disease as RhLCV also possesses a viral gene repertoire identical to EBV, immortalizes host B cells, and is associated with B cell lymphomas in immunocompromised hosts [21,25,26,52]. RhLCV-associated PTLD has been reported in three rhesus macaques undergoing renal transplantation [53]. However, development of a rhesus macaque model for EBV-associated lymphoproliferative disease in which disease can be reliably induced has proved challenging. Analogous to the study described here, Rivailler *et al*. attempted to induce LCV lymphomagenesis in RhLCV-naïve rhesus macaques using SHIV-mediated immunosuppression followed by autologous RhLCV-BLCL intravenous infusion [54]. Despite substantial SHIV-mediated CD4+ T cell depletion and successful RhLCV infection, only 1 of 4 rhesus macaques (25%) developed lymphoma. In contrast, we were able to induce a higher incidence of disease (4 of 5, 80%) in CyLCV-seropositive, SIV-infected Mauritian cynomolgus macaques using a combination of CD8α+ cell depletion and CyLCV-BLCL infusion, with and without additional immunosuppressants. Of note, previous studies documented a higher incidence of non-Hodgkin lymphoma in SIV-infected cynomolgus macaques compared to rhesus macaques [26,55], and thus our results are consistent with increased pathogenicity of CyLCV in cynomolgus macaques compared to RhLCV in rhesus macaques. However, it should be noted that all four LCV model MCMs that developed disease in the study described here received autologous CyLCV-BLCL created with a particular strain of CyLCV (derived from PTLD-experiencing HSCT recipient 35132 inguinal lymph node), and thus it is unclear if similar results can be obtained with other CyLCV strains. Due to their simplified MHC genetics [56], MCM models in particular would be advantageous for studying LCV-specific T cell responses and developing and testing T cell therapies for lymphocryptovirus-associated PTLD. A key limitation to this model is the inability to evaluate GVHD complications of PTLD treatments, such as those involving withdrawal or reduction in immunosuppression. However, this MCM model could also be useful to identify biomarkers that predict development of lymphocryptovirus-associated lymphoproliferative disease or disease severity among individuals as well as assess therapeutic efficacy and optimize timing of novel antiviral, chemotherapy, or other adoptive cell therapies. Therefore, our strategy for inducing CyLCV-associated lymphoproliferative disease without transplantation in MCMs represents a promising NHP model for testing new diagnostics, therapeutics, and management strategies for EBV-PTLD.

## Materials and methods

### Ethics statement

Mauritian-origin cynomolgus macaques (*Macaca fascicularis)* between 4–15 years of age at study initiation were housed at the ONPRC and utilized for studies under the approval of the Oregon Health and Science University (OHSU) West Campus Institutional Animal Care and Use Committee (IACUC). All macaques in this study were managed according to the ONPRC animal care program, which is fully accredited by AAALAC International and is based on the laws, regulations, and guidelines set forth by the United States Department of Agriculture (e.g., the Animal Welfare Act and Animal Welfare Regulations, the Guide for the Care and Use of Laboratory Animals, 8^th^ edition (Institute for Laboratory Animal Research), and the Public Health Service Policy on Humane Care and Use of Laboratory Animals. The nutritional plan utilized by the ONPRC is based on National Research Council recommendations and supplemented with a variety of fruits, vegetables, and other edible objects as part of the environmental enrichment program established by the Behavioral Services Unit. Animals were socially housed, when possible. All efforts were made to minimize suffering through the use of minimally invasive procedures, anesthetics, analgesics, and environmental enrichment. Where indicated, macaques were euthanized by exsanguination under a surgical plane of anesthesia induced by intravenous sodium pentobarbital followed by bilateral thoracotomy. The euthanasia method is approved by the American Veterinary Medical Association (AVMA Guidelines for the Euthanasia of Animals: 2020 Edition, https://olaw.nih.gov/policies-laws/avma-guidelines-2020.htm.

### Animal procedures

HSCT procedures for HSCT recipient MCMs 36481 and 36478 is previously described in detail [30,33]. Both MCMs 36481 and 36478 received 50 mg/kg SC dose of afucosylated anti-CD20 depleting antibody 2B8R1F8 (RRID: AB_2819341, engineered and produced by the NHP Reagent Resource). Complete drug regimens for each LCV model MCMs can be found in S2 Fig. All five LCV model MCMs received 4 doses of anti-CD8α depleting monoclonal antibody M-T807R1 (RRID: AB_2716320, engineered and produced by the NHP Reagent Resource), with 1 or 2 doses administered prior to infusion of autologous CyLCV-BLCL. The 4 anti-CD8α doses were administered as follows: #1 10 mg/kg SC, #2–4 5 mg/kg IV. Four LCV model MCMs received additional immunosuppression, including tacrolimus (0.06–0.1 mg/kg IM), belatacept (20 mg/kg IV), prednisone (2 mg/kg PO), dexamethasone (1 mg/kg IM), and/or anti-CD8β depleting monoclonal antibody CD8b255R1 (50 mg/kg SC, RRID: AB_2716321, engineered and produced by the NHP Reagent Resource). Daily tacrolimus doses were adjusted to maintain a tacrolimus trough level of 5–15 ng/ml in whole blood (measured by immunoassay, Abbott Architect i2000) and tapered as previously described [30].

### Bloodwork and blood and tissue processing

Macaque blood and tissues were collected and processed as previously described [33]. For complete blood counts, EDTA blood was run on an ABX Pentra 60C+ Hematology Analyzer. For plasma isolation, EDTA blood was centrifuged at 1860rcf for 10 minutes, and plasma was removed and clarified at 830rcf for 4 minutes. For peripheral blood mononuclear cell (PBMC) isolation, EDTA blood was layered on Ficoll-paque for density gradient centrifugation at 1860rcf for 30 min, and buffy coats containing mononuclear cells were removed and washed. Masses, lymph nodes, and spleen were diced and mashed through 70-micron cell strainers to collect single cell suspensions, and spleen was subsequently ACK-treated to lyse red blood cells. For bone marrow mononuclear cell preparations, bone marrow was pelleted, resuspended in PBS with 2mM EDTA with vigorous shaking, pelleted, and percoll gradient centrifugation as described below. For colon and jejunum, diced tissue pieces were incubated in R3 (RPMI160 with 3% fetal bovine serum) with 2mM EDTA for 30 minutes at 37C, shaking at 250rpm, and released cells were collected by straining samples thru a metal tea strainer (first collection). Residual tissue pieces were washed and then incubated in R3 with 0.2 mg/mL collagenase and 0.2 mg/mL DNase for 1 hour at 37C, shaking at 250 rpm. EDTA was added for a final concentration of 5 mM to inactivate digestion enzymes, and released cells were collected by straining samples thru a metal tea strainer (adding to the first collection). For liver and cerebellum, diced tissue pieces incubated in R3 with 0.2 mg/mL collagenase and 0.2 mg/mL DNase for 1 hour at 37C, shaking at 250 rpm (liver only). EDTA was added for a final concentration of 5 mM to inactivate digestion enzymes, and released cells were collected by mashing tissue through 70-micron cell strainers. Collections of bone marrow, colon, jejunum, liver, and cerebellum cells were pelleted and resuspended in 70% isotonic Percoll, underlayed below 37% isotonic Percoll, and centrifuged at 500rcf for 20 minutes. Buffy coats containing mononuclear cells were collected and washed.

### Antiretroviral therapy and viral detection assays

This study includes 2 HSCT recipient macaques and 5 LCV model macaques, all seropositive for LCV by BFRF3 sVCA ELISA [57] and all infected with SIVmac239 in previous studies [33,58]. HSCT recipient macaques were SIVmac239-infected for ~5 months prior to HSCT; LCV model macaques were SIVmac239-infected for 6–12 months prior to CyLCV-BLCL infusion. The two HSCT recipient macaques (36481 and 36478) and one LCV model macaque (38141) received daily combination antiretroviral therapy (ART) administered in a once-daily subcutaneous injection and consisting of tenofovir disoproxil fumarate (TDF; 5.1 mg/kg), emtricitabine (FTC; 40 mg/kg), and dolutegravir (DTG; 2.5 mg/kg) initially provided by Gilead and ViiV Healthcare, and subsequently purchased from APIChem and formulated as described [59]. SIV viral plasma loads were measured as previously described [33] with a limit of quantification (LOQ) of 50 copies viral RNA per mL plasma. Plasma, supernatant, and cell-associated CyLCV viral loads were measured as previously described [30]. Briefly, viral nucleic acid was extracted from plasma or culture supernatant using QIAamp MinElute Virus Spin kit. Total cellular DNA was isolated from cells using Qiagen DNeasy kit (Qiagen, Venlo, Netherlands) according to manufacturer’s instructions. Duplex quantitative PCR for CyLCV EBER-1 and cynomolgus macaque albumin (to measure cell input for cell-associated viral loads) was performed in duplicate alongside no template control reactions and standard reactions containing ten-fold serial dilutions of standard (1 copy to 10^7^ copies per reaction, in duplicate). Plasmids (pCEP4) containing each target region served as standards. Plates were run on a StepOnePlus Real-Time PCR system (Applied Biosystems) with the following conditions: 95°C 00:20, followed by 40 cycles of 95°C 00:01, 60°C 00:20. Target DNA copy numbers in each sample reaction were calculated according to the standard curve. For plasma and supernatant CyLCV viral loads, quantities were adjusted for the proportion of viral nucleic acid added to the reaction and initial sample volume to obtain CyLCV viral DNA copies per milliliter, and the assay LOQ is indicated in each Fig legend (60 copies per mL or 300 copies per mL). For cell-associated viral loads, cell input into each reaction was calculated by dividing albumin copy number by 2, and cell-associated viral load (copies per million cells) was calculated by dividing the CyLCV reaction copy number by the reaction cell input, then multiplying by 1×10^6^. LOQ for cell-associated viral loads varied slightly among assays due to variable cell input, but was equal to or lower than 260 copies per million cells.

### Cell sorting and culture

Where indicated, CD20+ cells were isolated by magnetic isolation with anti-FITC microbeads (Miltenyi) after staining with CD20 (clone 2H7, FITC, BD), according to manufacturer’s instructions. For primary cell cultures from biopsy and necropsy tissues, processed single cell suspensions or isolated CD20+ cells were plated in R10 (RPMI1640, 10% fetal bovine serum, antibiotic/antimycotic (HyClone), 2mM L-glutamine, 1mM sodium pyruvate). Cultures were maintained in R10, replacing media and splitting cells as culture medium yellowed. To generate new CyLCV-BLCLs *ex vivo*, isolated CD20+ cells from PBMC of indicated MCMs were plated at 100,000 cells per well of U-bottom 96-well plates in 100μL of R20 (R10 but with 20% fetal bovine serum) and 100μL of clarified, 0.22μm-filtered CyLCV-containing supernatant from the indicated culture of primary CyLCV+ BLCL expanded from CyLCV-PTLD-experiencing HSCT recipient MCM tissues. Compared to stagnant control wells plated with plain R20, CyLCV+ supernatant-containing wells expanded quickly into large pellets and yellowed the culture medium. *Ex vivo* CyLCV-BLCL were gradually expanded and transitioned into R10. For the LCV model study, the indicated autologous CyLCV-BLCLs (Fig 2C) were aliquoted from cultures, washed with PBS, filtered through a 70-micron cell strainer, resuspended in 10mL PBS, loaded into luer-lok syringes, and infused into LCV model study MCMs. For culture cell-associated and supernatant CyLCV measurements, cells were washed then plated at 1x10^6^ per mL R10 for seven days. After seven days, cultures were harvested and pelleted at 830rcf for 4 min, supernatant was removed and clarified at 830rcf for 4min, and cells were washed with R10. Nucleic acids were extracted from clarified supernatants and washed cell pellets and measured for CyLCV by qPCR as described above (see Antiretroviral therapy and viral detection assays section).

### Flow cytometry phenotyping and CFSE proliferation assays

Blood and tissue lymphocyte frequencies and intracellular Ki67+ levels were monitored by flow cytometric staining as previously described [33,60]. Briefly, EDTA-treated whole blood (50–100 μL) or single cell suspensions were washed twice with PBS and stained for surface markers and viability (Live/dead Yellow Fixable, Invitrogen) at room temperature for 30 min, resuspended in 1 mL 1X FACS Lysing Solution (BD) to lyse red blood cells and fix remaining cells, incubated at room temperature for 8 min, and washed three times with FACS buffer (1X PBS with 10% bovine growth serum). For intracellular staining, samples were then permeabilized with 500 μL of FACSperm (1X FACS Lysing Buffer with 0.05% Tween-20), incubated at room temperature for 10 min, washed three times with FACS buffer, stained for intracellular markers at room temperature for 45 min, and washed once with FACS buffer. Stained samples were collected on a BD LSRII instrument, and flow cytometric analysis was performed using Flow Jo (BD). Three flow cytometry staining panels were utilized for staining *ex vivo* samples. Staining panel 1 (Figs 1C, 1D, 3B, 3C, 3D, S1B, S1C and S6A) included surface staining for CD45 (clone D058-1283, APC, BD), CD3 (clone SP34-2, Pacific Blue, BD), CD20 (clone 2H7, FITC, BD), and Live/dead fixable yellow, and intracellular staining for Ki67 (clone B56, PerCP-Cy5.5, BD) and CD79a (clone HM47, APC-eFluor780, eBioscience). Staining panel 2 (S4A and S4B Fig CD8α+ T cells, CD8α+ NK cells, CD20+ B cells) included surface staining for CD45 (clone D058-1283, APC, BD), CD3 (clone SP34-2, Alexa 700, BD), CD20 (clone 2H7, APC-H7, BD), CD4 (clone L200, PerCP-Cy5.5, BD), CD8α (clone DK25, Pacific Blue, Dako), and Live/dead fixable yellow (Life Technologies). Staining panel 3 (S4B Fig CD8β+ T cells) included surface staining for CD45 (clone D058-1283, APC, BD), CD3 (clone SP34-2, Pacific Blue, BD), CD20 (clone 2H7, FITC, BD), CD4 (clone L200, PerCP-Cy5.5, BD), CD8α (clone SK1, APC-H7, BD), CD8β (clone 2ST8.5H7, PE, Beckman Coulter), and Live/dead fixable yellow. Unlike clone SK1, anti-CD8α clone DK25 in staining panel 2 can resolve CD8α+ cells in the presence of depleting antibody M-T807R1 [61,62]. Cell subsets within the live CD45+ gate were defined as follows: CD20+ B cells (CD3-, CD20+), CD79a+ B cells (CD3-, CD79a+), CD4+ T cells (CD3+, CD20-, CD4+), CD8α+ T cells (CD3+, CD20-, clone DK25 CD8α+), CD8β+ T cells (CD3+, CD20-, CD8β+), CD8α+ NK cells (CD3-, CD20-, clone DK25 CD8α+). Note *in vivo* anti-CD20 treatment blocks anti-CD20 clone 2H7 binding, and thus CD79a+ B cells were also monitored. Absolute lymphocyte counts in blood were calculated by multiplying whole blood staining frequencies within the live CD45+ gate by the white blood cell count (WBC), as determined by complete blood count performed on an additional aliquot of EDTA-treated whole blood drawn at the same time.

For LCV-BLCL phenotyping (S3A Fig), LCV-BLCL cultures or thawed PBMC were surface stained with amine-reactive dye (Invitrogen live/dead fixable near-IR) and antibodies against CD20 (clone 2H7, FITC, BD) and fixed with 2% PFA. Stained samples were collected on a BD FACSymphony A5 instrument, and flow cytometric analysis was performed using Flow Jo (BD). For CFSE proliferation assays (S3B Fig), cells were labeled with 1μM carboxyfluorescein succinimidyl ester (CFSE), washed thoroughly with cold R10, then plated in triplicate in warm R10 at 100,000 cells per well of 96-well U-bottom plates. At days 3 and 7, half of the cells from each culture were harvested and surface stained with amine-reactive dye (Invitrogen live/dead fixable near-IR) and fixed with 2% PFA. Stained samples were collected on a BD FACSymphony A5 instrument, and flow cytometric analysis was performed using Flow Jo (BD). For each sample, the CFSE geometric mean MFIs of the three replicates (gated on live cells) were averaged to obtain an average CFSE geometric mean MFI for that sample at that timepoint. To assess proliferation, fold change was calculated by dividing the average day 7 CFSE geometric mean MFI by the average day 3 CFSE geometric mean MFI for each sample.

### Histology and immunohistochemistry

Representative samples of major organs and lymph nodes were examined and collected in 10% neutral buffered formalin. Tissues for microscopic examination were processed, embedded in paraffin, and sectioned at 5 μm for histology. Tissue collection, gross necropsy and microscopic examinations were performed by three board-certified veterinary pathologists (A.L.J.; L.C.; A.D.L.). H&E-stained tissue sections were evaluated and lymphomas and plasma cell hyperplasia were identified. Lymphomas were characterized by sheets of monomorphic round cells supported by a fine fibrovascular stroma. Neoplastic cells were relatively large, uniform, round to oval with moderate amounts of eosinophilic cytoplasm. Nuclei were round to oval with one to two large eosinophilic nucleoli per nucleus and clumped chromatin. There was mild to moderate anisocytosis. Mitotic rate was brisk. Neoplastic lymphocytes invaded and effaced normal architecture in the affected tissues. Plasma cell hyperplasia was characterized by high numbers of mature plasma cells within medullary cords of multiple lymph nodes which were not associated with sites of chronic inflammation. Additional slides were stained for B cell marker CD20 and EBNA2 via immunohistochemistry. Immunohistochemistry was performed on the intelliPATH FLX automated slide stainer (Biocare Medical, Pacheco, CA). Deparaffinized slides underwent antigen retrieval in citrate buffer (pH 6.0) using the Decloaking Chamber (Biocare Medical, Pacheco, CA). Slides were then double-stained with the mouse primary anti-EBNA2 antibody (EBNA2) (clone PE2, Ab90543, Abcam Inc., Cambridge, MA) and the goat primary anti-CD20 antibody (PA1-9024, Invitrogen), both diluted at a 1:500 dilution with 0.25% casein in TBS-T, for one hour at room temperature. Following this, slides were incubated with 3% Hydrogen peroxide for 10 minutes at room temperature. Next, the GBI Polink 2 Polymer HRP anti-mouse IgG (Origene, Rockville, MD) was applied to the slides for 20 minutes at room temperature, followed by the GBI Polink 1 AP anti-goat IgG for 20 minutes at room temperature. EBNA2 was then visualized with Deep Space Black chromgen kit (Biocare Medical). CD20 was visualized with Warp Red (Biocare Medical) and counterstained with hematoxylin.

### Whole genome CyLCV sequencing

For viral supernatant preparation and viral DNA extraction, CyLCV-BLCL cultures were expanded to >100 million cells, plated at 3 million cells/mL density in T175 flasks, treated with 50ng/mL 12-O-Tetradecanoylphorbol-13-acetate and 3mM sodium butyrate for 18–24 hours, then pelleted, washed, re-plated with fresh R10, and incubated for 5–7 days before supernatants were collected, filtered, and purified via ultra-centrifugation. Virion DNA was extracted using Wizard DNA Purification Kit (Promega) according to manufacturer’s instructions. 2μg of extracted DNA was subject to library preparation with Illumina DNA prep kit and sequenced on Illumina iSeq according to manufacturer’s instructions. Paired-end 150 nucleotide reads were generated using the iSeq. Reads were obtained in fastq format and sequence quality initially assessed using FastQC (Galaxy Version 0.74). CyLCV genome sequence reads were subsequently mapped with Bowtie2 (Galaxy Version 2.5.3) (Langmead, 2012) using default parameters and Lymphocryptovirus Macaca/pfe-lcl-E3 (GenBank accession number NC_055142) as the reference genome. Read coverage for each sample was visualized using Geneious Prime v2023.0.4. All samples contained intact, full length CyLCV viral genomes with no major insertions or deletions (Genbank accession numbers PQ385619, PQ385620, PQ385621, PQ385622, PQ385623).

### 18F-FDG PET/CT

All PET scans were performed on a Discovery MI 710–64 slice PET/CT scanner (GE Healthcare). 18F-FDG (3.37mCi-3.86mCi, mean dose of 3.62mCi) was obtained from a local commercial radiopharmacy and injected 60 minutes prior imaging. PET images were obtained over 6 bed positions (10% overlap) at 5 minutes per bed to include the whole body (vertex to foot). A helical CT scan was performed prior to PET imaging (120kV, 225mA, 0.625mm slices and 3.75mm slices for CTAC). The images were analyzed on MIM software (MIM Software, Inc., version 7.2.6, Cleveland, Ohio). Regions of interest (ROIs) were placed, by one experienced Nuclear Medicine physician (E.M.), over the various organs and lesions of interest to calculate a standardized uptake value (SUV) on each of the scans throughout the experiment period.

## Supporting information

S1 Fig Additional data from two HSCT recipient MCMs with CyLCV-PTLD. **(A)** MCM percent weight change relative to pre-HSCT. Colored crosses next to datapoints indicate time of euthanasia/death for each MCM. **(B, C)** Summary graphs of flow cytometry staining of MCM 36481 necropsy tissues (B) and 36478 excised submandibular lymph node mass and necropsy tissues (C). Frequencies of CD20+ B cells, CD79a+ B cells, CD3+ T cells among live CD45+ cells (left). Frequencies of Ki67+ cells among live CD20+ B cells, CD79a+ B cells, CD3+ T cells (right). White patterned bars denote affected tissues positive for lymphoma. Asterisks (*) indicate Ki67+ frequencies not shown due to low B cell frequency (<2% of live CD45+ cells). **(D)** Representative images of MCM 36481 B cell lymphoma at necropsy. Upper left: Gross photograph of small intestine and associated mesenteric lymph nodes (marker = 1 cm). The mesenteric lymph nodes are severely enlarged. A 3cm segment of the ileum is firm and enlarged. Upper right: H&E staining of small intestine tissue section (marker = 1mm). Lymphoma arising from the submucosal gut-associated lymphoid tissue effaces normal microarchitecture and infiltrates the overlying mucosa. Lower left: H&E staining of small intestine tissue section (marker = 50 μm). A monomorphic population of neoplastic lymphocytes effaces normal microarchitecture. Lower right: Dual immunohistochemistry staining of small intestine tissue section for CD20 (Warp red) and lymphocryptovirus EBNA2 (deep space black) (marker = 50 μm). The majority of neoplastic lymphocytes are immunoreactive for CD20 (membrane and cytoplasm) and EBNA2 (nuclear), indicating B cell lineage lymphocytes infected with LCV. **(E)** Cell-associated (left) and supernatant (right) CyLCV DNA viral loads in primary cultures of tissue single cell suspensions from PTLD-experiencing HSCT recipients (primary LCV-BLCL). Bars show mean ±SD of two qPCR replicates. TOD = time of death, AxLN = axillary lymph node, IngLN = inguinal lymph node, MesLN = mesenteric lymph node, SmLN = submandibular lymph node.
(TIF)

S2 Fig Drug regimens for LCV model MCMs. SC = subcutaneous, IV = intravenous, IM = intramuscular, PO = per os (oral), ART = combination antiretroviral therapy. MHC types for each MCM are indicated at the top of each table.
(TIF)

S3 Fig Characterizing LCV-BLCL lines. **(A)** Representative flow cytometry plots show surface CD20 of infused LCV-BLCL lines, primary LCV-BLCL from 35132 IngLN and 36478 SmLN, and control *ex vivo* MCM PBMC from 34667 prior to study. MCM PBMC was utilized to set the gate determining positive CD20 staining for LCV-BLCL lines. Plots are gated on live singlets. **(B)** CFSE proliferation assay of cells from (A). Graphs show fold change in CFSE geometric mean MFI of live cells between day 3 and day 7 after CFSE labeling (average of three culture replicates). IngLN = inguinal lymph node, SmLN = submandibular lymph node.
(TIF)

S4 Fig Additional longitudinal data from LCV model MCMs. **(A)** Longitudinal plasma SIVmac239 RNA viral loads (top) and absolute counts of CD4+ T cells in blood (bottom). Drug regimens and CyLCV-BLCL infusion timepoints indicated above graphs with colors corresponding to each MCM shown in graphs. Plasma viral load LOQ = 50 copies/mL. Undetectable or below LOQ measurements are graphed at the LOQ. **(B)** Longitudinal absolute counts of CD8α+ T cells, CD8β+ T cells, CD8α+ NK cells, and CD20+ B cells in blood. **(C)** MCM percent weight change relative to study baseline (prior to first anti-CD8α dose).
(TIF)

S5 Fig Representative images of MCM 37053 B cell lymphoma at necropsy. Upper left: Gross photograph of stomach (marker = 1 cm). Multiple raised mucosal masses in the cardia and fundus; several exhibit central umbilication (necrosis). Upper right: H&E staining of stomach tissue section (marker = 1mm). Neoplastic lymphocytes diffusely infiltrate and partially efface all layers of the stomach. Lower left: H&E staining of stomach tissue section (marker = 50 μm). Monomorphic population of neoplastic lymphocytes effaces normal gastric architecture. Lower right: Dual immunohistochemistry staining of stomach tissue section for CD20 (Warp red) and lymphocryptovirus EBNA2 (deep space black) (marker = 50 μm). Majority of neoplastic lymphocytes are immunoreactive for CD20 (membrane and cytoplasm) and EBNA2 (nuclear), indicating B cell lineage lymphocytes infected with LCV.
(TIF)

S6 Fig Additional data from necropsies of LCV model MCMs. **(A)** Summary graphs of flow cytometry staining of model MCM necropsy tissues. Frequencies of CD3+ T cells among live CD45+ cells (left). Frequencies of Ki67+ cells among live CD3+ T cells (right). Bars not shown indicate samples not assayed. White patterned bars denote affected tissues positive for lymphoma. **(B)** Cell-associated (left) and supernatant (right) CyLCV DNA viral loads in primary cultures of necropsy tissue CD20+ cells. White patterned bars denote affected source tissues positive for lymphoma. Bars show mean ±SD of two qPCR replicates. Limit of quantification (LOQ) = 260 copies/million cells for cell-associated; 300 copies/mL for supernatant. Undetectable and below LOQ measurements graphed at the LOQ. AxLN = axillary lymph node, IngLN = inguinal lymph node, MesLN = mesenteric lymph node, PancrLN = pancreatic lymph node, IleocLN = ileocecal lymph node, HepLN = hepatic LN.
(TIF)
