# Supplementary figures and images for: A model of lymphocryptovirus-associated post-transplant lymphoproliferative disorder in immunosuppressed Mauritian cynomolgus macaques
Source: PLoS Pathog. 2024 Nov 11;20(11):e1012644. doi: 10.1371/journal.ppat.1012644 (PMC11581395; doi:10.1371/journal.ppat.1012644)

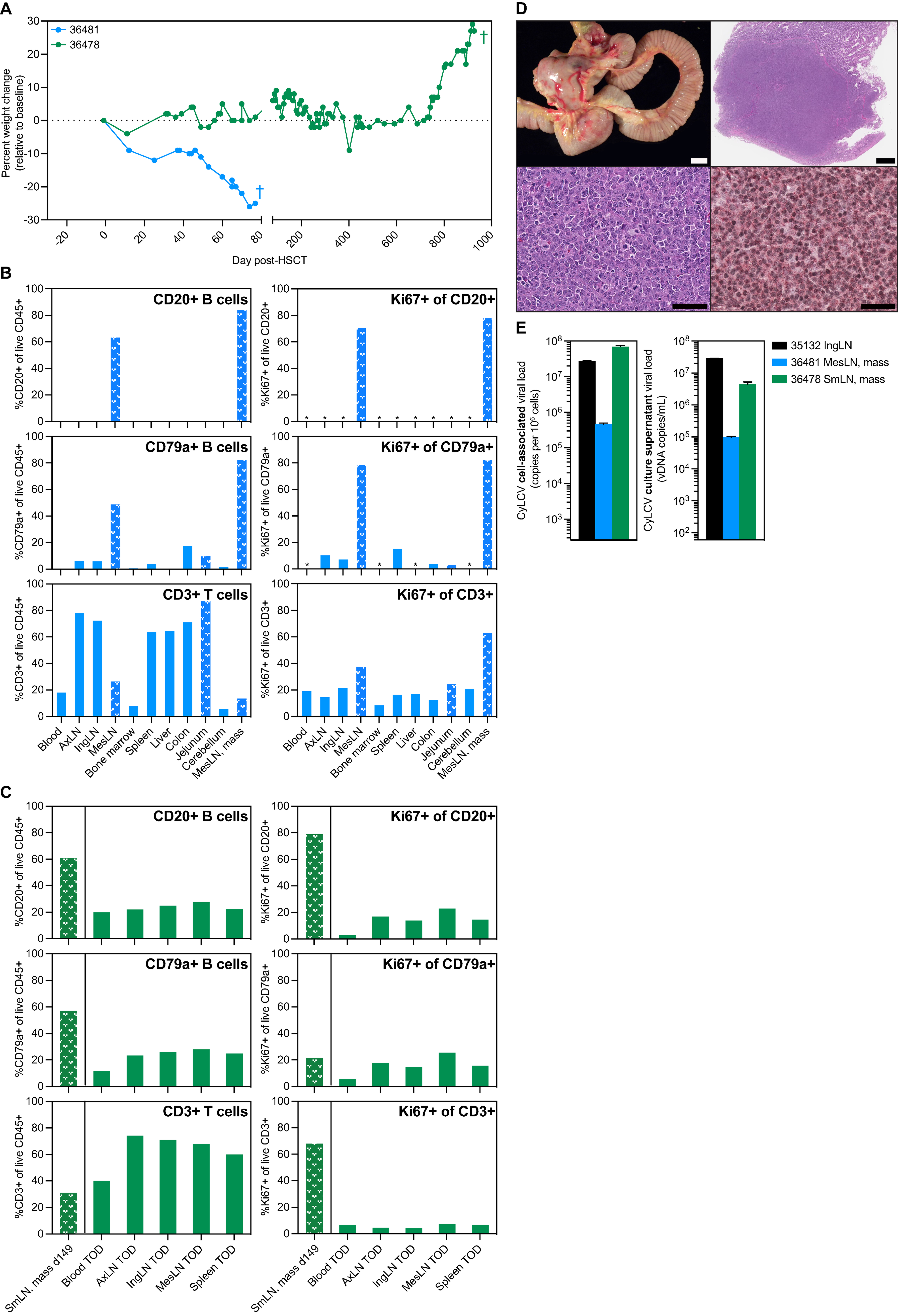

Supplement: S1 Fig — (A) MCM percent weight change relative to pre-HSCT. Colored crosses next to datapoints indicate time of euthanasia/death for each MCM. (B, C) Summary graphs of flow cytometry staining of MCM 36481 necropsy tissues (B) and 36478 excised submandibular lymph node mass and necropsy tissues (C). Frequencies of CD20+ B cells, CD79a+ B cells, CD3+ T cells among live CD45+ cells (left). Frequencies of Ki67+ cells among live CD20+ B cells, CD79a+ B cells, CD3+ T cells (right). White patterned bars denote affected tissues positive for lymphoma. Asterisks (*) indicate Ki67+ frequencies not shown due to low B cell frequency (<2% of live CD45+ cells). (D) Representative images of MCM 36481 B cell lymphoma at necropsy. Upper left: Gross photograph of small intestine and associated mesenteric lymph nodes (marker = 1 cm). The mesenteric lymph nodes are severely enlarged. A 3cm segment of the ileum is firm and enlarged. Upper right: H&E staining of small intestine tissue section (marker = 1mm). Lymphoma arising from the submucosal gut-associated lymphoid tissue effaces normal microarchitecture and infiltrates the overlying mucosa. Lower left: H&E staining of small intestine tissue section (marker = 50 μm). A monomorphic population of neoplastic lymphocytes effaces normal microarchitecture. Lower right: Dual immunohistochemistry staining of small intestine tissue section for CD20 (Warp red) and lymphocryptovirus EBNA2 (deep space black) (marker = 50 μm). The majority of neoplastic lymphocytes are immunoreactive for CD20 (membrane and cytoplasm) and EBNA2 (nuclear), indicating B cell lineage lymphocytes infected with LCV. (E) Cell-associated (left) and supernatant (right) CyLCV DNA viral loads in primary cultures of tissue single cell suspensions from PTLD-experiencing HSCT recipients (primary LCV-BLCL). Bars show mean ±SD of two qPCR replicates. TOD = time of death, AxLN = axillary lymph node, IngLN = inguinal lymph node, MesLN = mesenteric lymph node, SmLN = submandibular lymph node [file ppat.1012644.s001.tif]

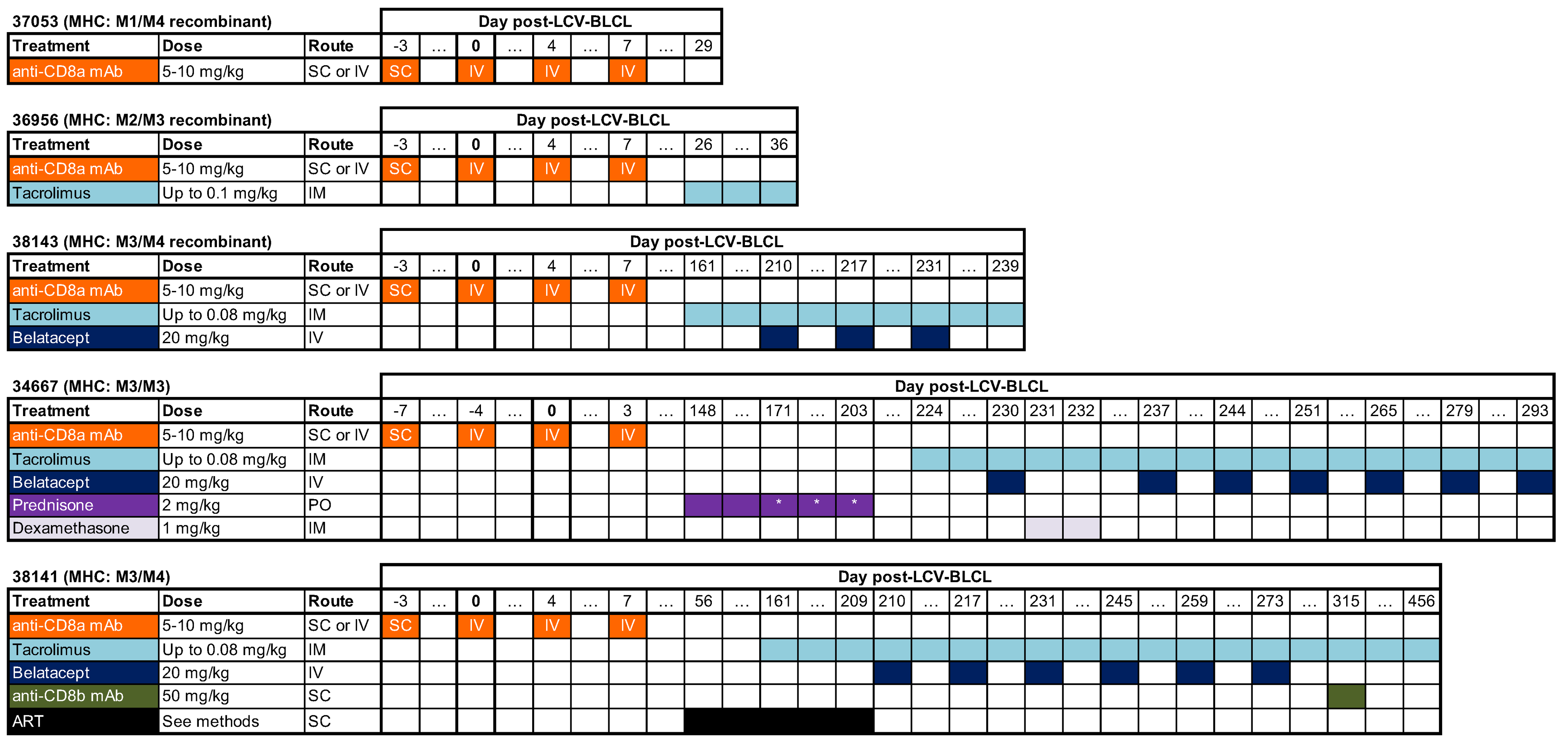

Supplement: S2 Fig — SC = subcutaneous, IV = intravenous, IM = intramuscular, PO = per os (oral), ART = combination antiretroviral therapy. MHC types for each MCM are indicated at the top of each table. (TIF) [file ppat.1012644.s002.tif]

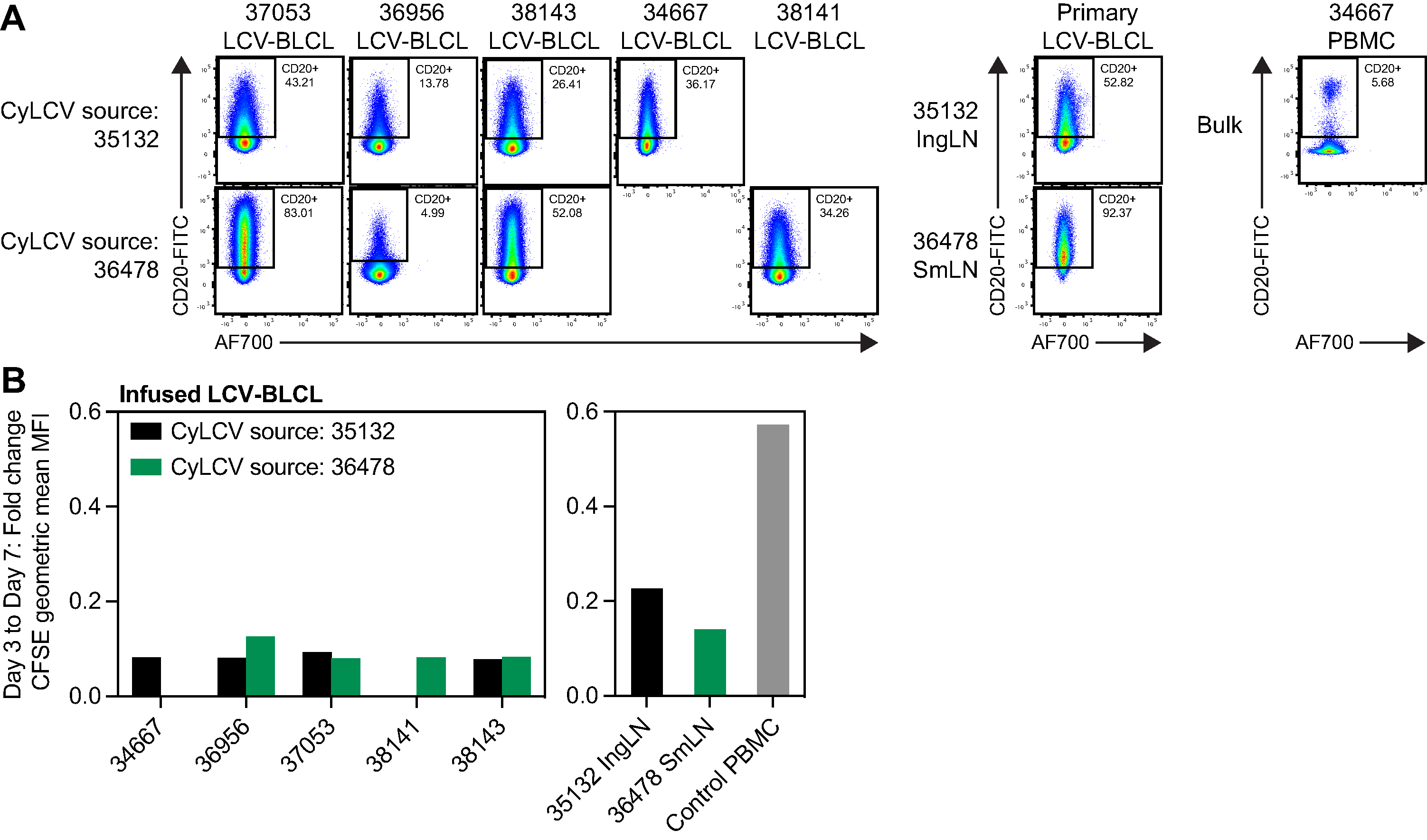

Supplement: S3 Fig — (A) Representative flow cytometry plots show surface CD20 of infused LCV-BLCL lines, primary LCV-BLCL from 35132 IngLN and 36478 SmLN, and control ex vivo MCM PBMC from 34667 prior to study. MCM PBMC was utilized to set the gate determining positive CD20 staining for LCV-BLCL lines. Plots are gated on live singlets. (B) CFSE proliferation assay of cells from (A). Graphs show fold change in CFSE geometric mean MFI of live cells between day 3 and day 7 after CFSE labeling (average of three culture replicates). IngLN = inguinal lymph node, SmLN = submandibular lymph node. (TIF) [file ppat.1012644.s003.tif]

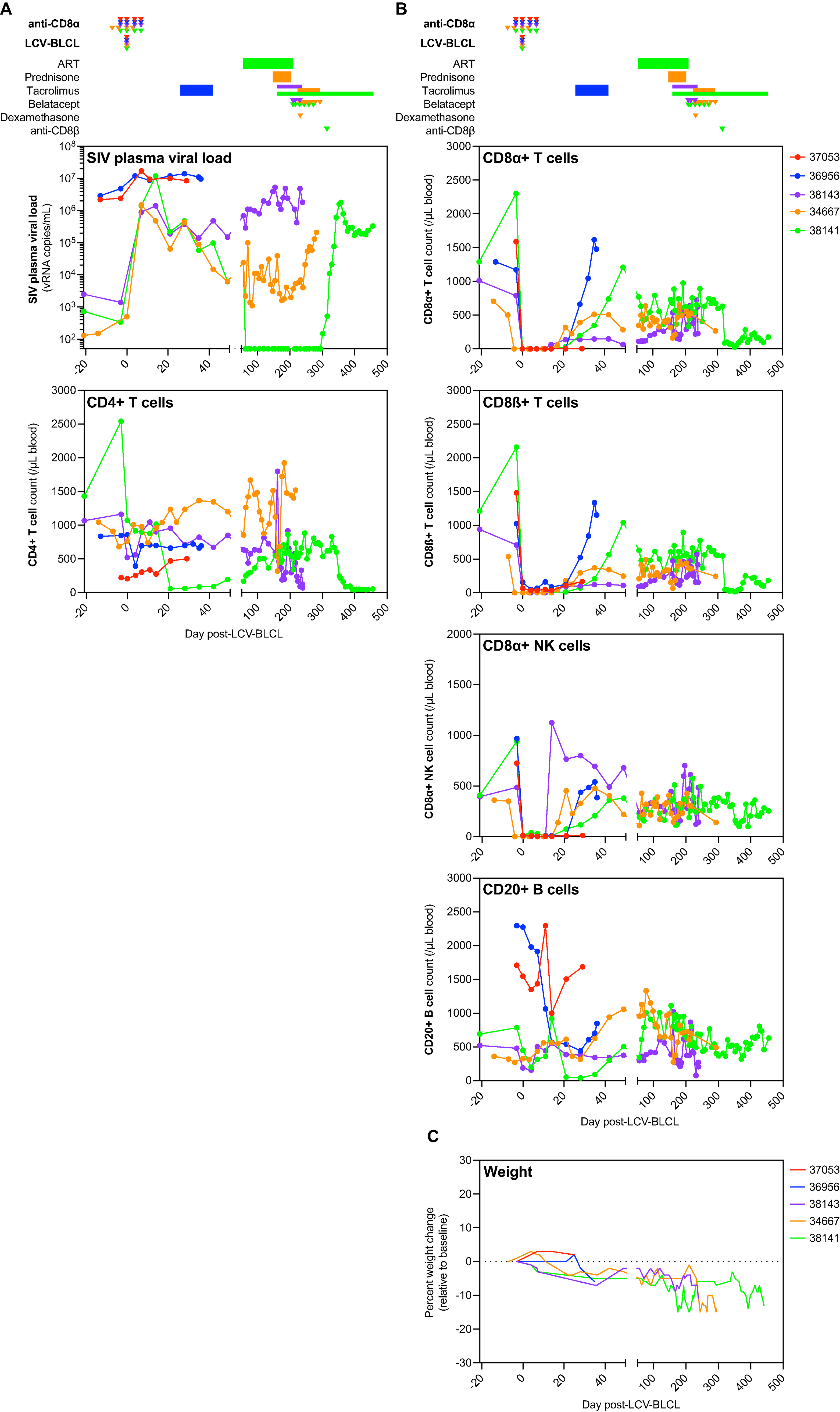

Supplement: S4 Fig — (A) Longitudinal plasma SIVmac239 RNA viral loads (top) and absolute counts of CD4+ T cells in blood (bottom). Drug regimens and CyLCV-BLCL infusion timepoints indicated above graphs with colors corresponding to each MCM shown in graphs. Plasma viral load LOQ = 50 copies/mL. Undetectable or below LOQ measurements are graphed at the LOQ. (B) Longitudinal absolute counts of CD8α+ T cells, CD8β+ T cells, CD8α+ NK cells, and CD20+ B cells in blood. (C) MCM percent weight change relative to study baseline (prior to first anti-CD8α dose). (TIF) [file ppat.1012644.s004.tif]

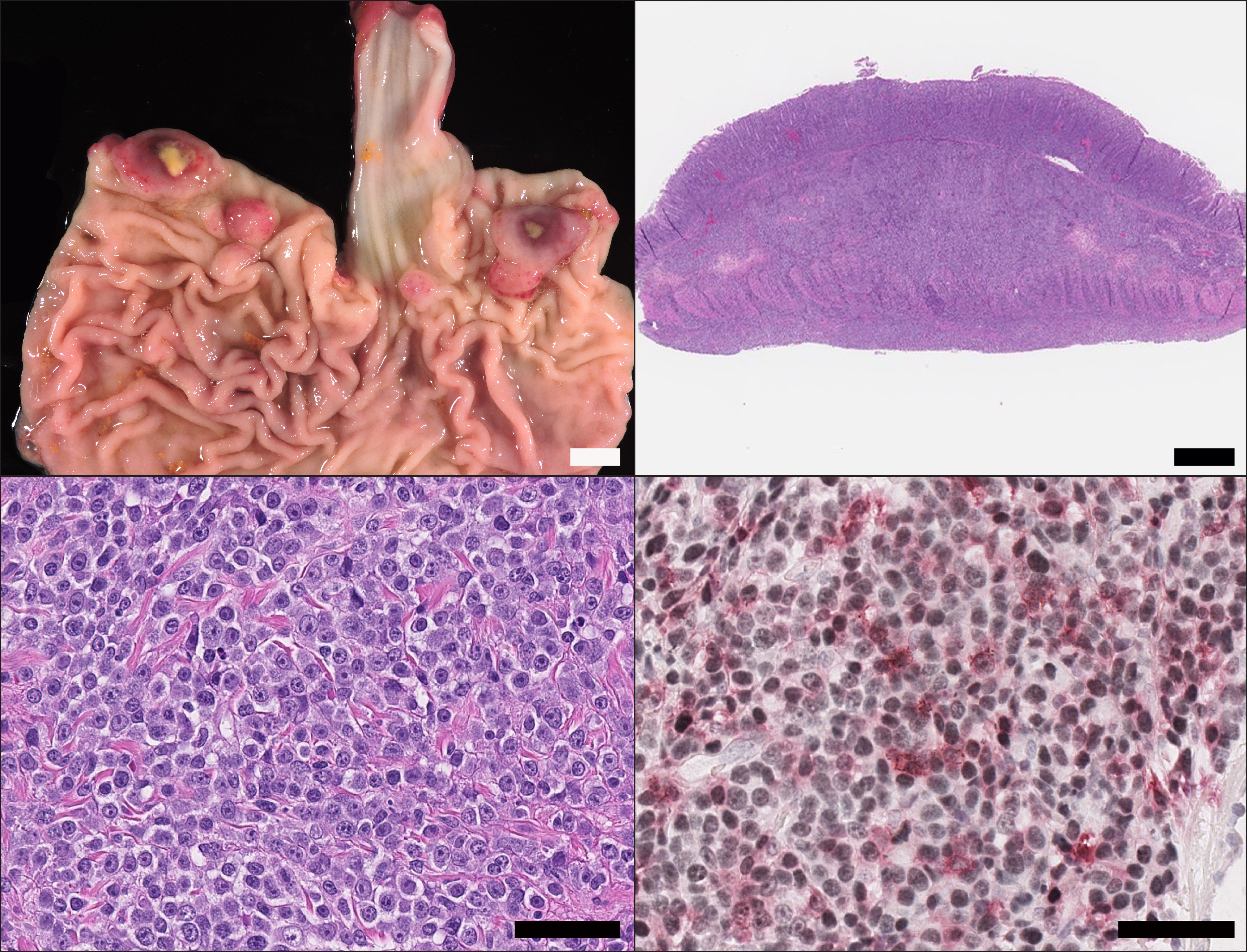

Supplement: S5 Fig — Upper left: Gross photograph of stomach (marker = 1 cm). Multiple raised mucosal masses in the cardia and fundus; several exhibit central umbilication (necrosis). Upper right: H&E staining of stomach tissue section (marker = 1mm). Neoplastic lymphocytes diffusely infiltrate and partially efface all layers of the stomach. Lower left: H&E staining of stomach tissue section (marker = 50 μm). Monomorphic population of neoplastic lymphocytes effaces normal gastric architecture. Lower right: Dual immunohistochemistry staining of stomach tissue section for CD20 (Warp red) and lymphocryptovirus EBNA2 (deep space black) (marker = 50 μm). Majority of neoplastic lymphocytes are immunoreactive for CD20 (membrane and cytoplasm) and EBNA2 (nuclear), indicating B cell lineage lymphocytes infected with LCV. (TIF) [file ppat.1012644.s005.tif]

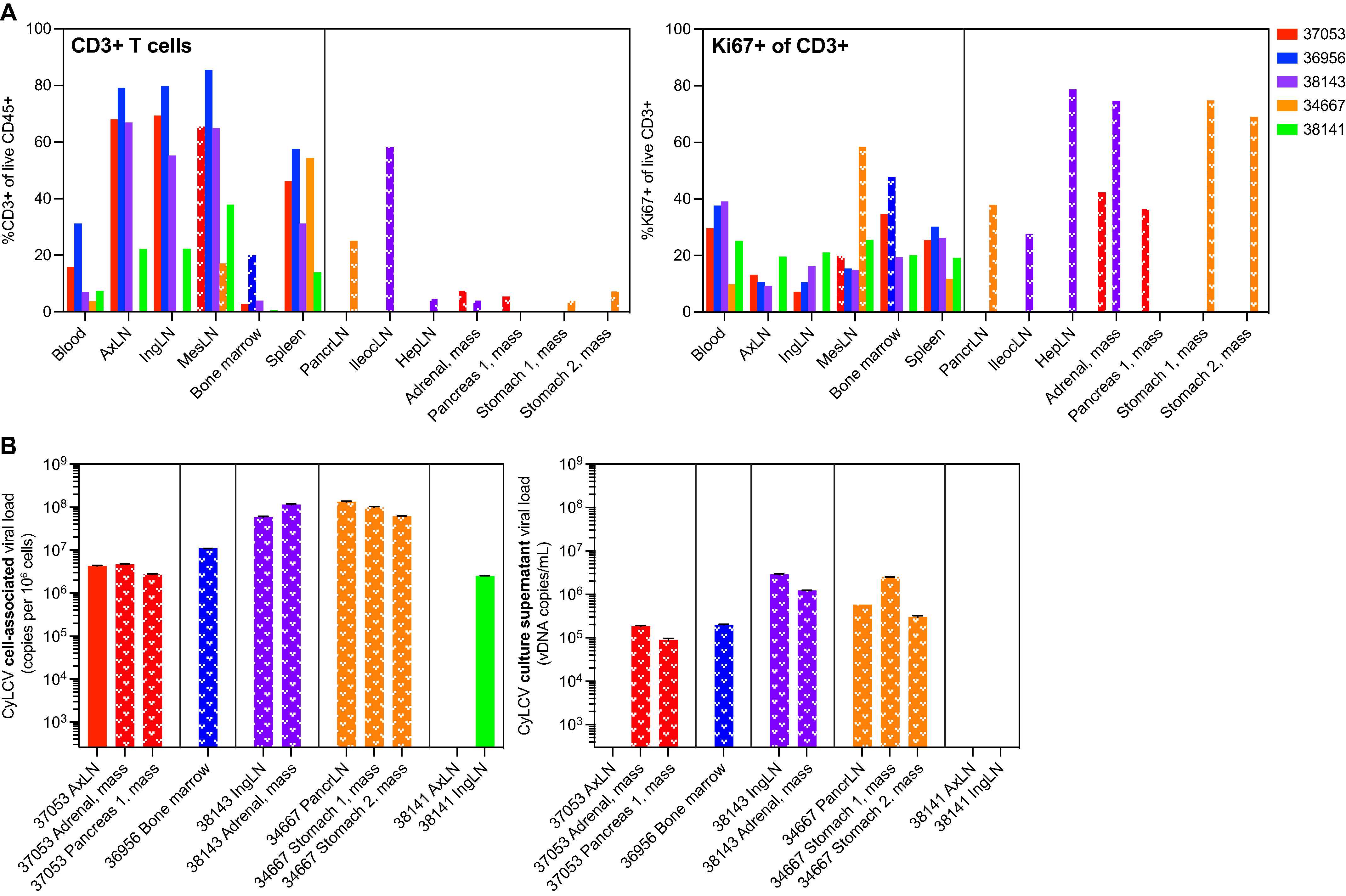

Supplement: S6 Fig — (A) Summary graphs of flow cytometry staining of model MCM necropsy tissues. Frequencies of CD3+ T cells among live CD45+ cells (left). Frequencies of Ki67+ cells among live CD3+ T cells (right). Bars not shown indicate samples not assayed. White patterned bars denote affected tissues positive for lymphoma. (B) Cell-associated (left) and supernatant (right) CyLCV DNA viral loads in primary cultures of necropsy tissue CD20+ cells. White patterned bars denote affected source tissues positive for lymphoma. Bars show mean ±SD of two qPCR replicates. Limit of quantification (LOQ) = 260 copies/million cells for cell-associated; 300 copies/mL for supernatant. Undetectable and below LOQ measurements graphed at the LOQ. AxLN = axillary lymph node, IngLN = inguinal lymph node, MesLN = mesenteric lymph node, PancrLN = pancreatic lymph node, IleocLN = ileocecal lymph node, HepLN = hepatic LN. (TIF) [file ppat.1012644.s006.tif]
